# Automating microsatellite screening and primer design from multi-individual libraries using Micro-Primers

**DOI:** 10.1038/s41598-021-04275-8

**Published:** 2022-01-07

**Authors:** Filipe Alves, Filipa M. S. Martins, Miguel Areias, Antonio Muñoz-Mérida

**Affiliations:** 1 Faculty of Sciences, University of Porto, Porto, Portugal; 2 CIBIO, InBIO-Research Network in Biodiversity and Evolutionary Biology, Porto, Portugal

**Keywords:** Computational biology and bioinformatics, Population genetics, Sequencing

## Abstract

Analysis of intra- and inter-population diversity has become important for defining the genetic status and distribution patterns of a species and a powerful tool for conservation programs, as high levels of inbreeding could lead into whole population extinction in few generations. Microsatellites (SSR) are commonly used in population studies but discovering highly variable regions across species’ genomes requires demanding computation and laboratorial optimization. In this work, we combine next generation sequencing (NGS) with automatic computing to develop a genomic-oriented tool for characterizing SSRs at the population level. Herein, we describe a new Python pipeline, named Micro-Primers, designed to identify, and design PCR primers for amplification of SSR loci from a multi-individual microsatellite library. By combining commonly used programs for data cleaning and microsatellite mining, this pipeline easily generates, from a fastq file produced by high-throughput sequencing, standard information about the selected microsatellite loci, including the number of alleles in the population subset, and the melting temperature and respective PCR product of each primer set. Additionally, potential polymorphic loci can be identified based on the allele ranges observed in the population, to easily guide the selection of optimal markers for the species. Experimental results show that Micro-Primers significantly reduces processing time in comparison to manual analysis while keeping the same quality of the results. The elapsed times at each step can be longer depending on the number of sequences to analyze and, if not assisted, the selection of polymorphic loci from multiple individuals can represent a major bottleneck in population studies.

## Introduction

At the Omics’ era, the cost of sequencing and time required for getting useful information from different organisms, even uncultured, has been drastically reduced with the advances in technology^1^, which allowed the broadening of its scientific application worldwide. While traditional studies covered a gene region and/or a pathway with limited number of genes, next generation sequencing (NGS) has pushed the trend towards whole-genome analysis and population genetics, where the genome of several individuals of a species can be characterized at the same time^2^. In this field, molecular techniques such as genotyping by sequencing (GBS) and marker-assisted selection (MAS) have gained prominence for not requiring a reference genome^3,4^ but also due to the possibility of characterizing a whole species at a lower cost, providing comprehensive information for both evolution studies and conservation efforts^5,6^.

Genetic polymorphisms, such as single nucleotide polymorphisms (SNPs) or simple sequence repeats (SSRs), also known as microsatellites, have served the field of population genetics^7,8^. SSRs are repeated DNA motifs that occur in non-coding regions, evenly distributed throughout the genome. They are excellent markers for genotype identification, genetic diversity, and genetic-phenotype mapping, at both species and population levels, due to their high levels of polymorphism^9,10^. Traditional methods normally use a single individual per species for microsatellite library development. The number of microsatellite loci genotyped afterwards needs to be limited as a balance between the cost associated with microsatellite design and optimization, since the microsatellite found for a single individual may not be polymorphic in the population so the efforts in sequencing afterwards could be in vain. In this work, we aimed to design and implement an automated pipeline for screening polymorphic SSRs using as input raw reads from a multi-individual sample. The tool can detect the variation in SSRs loci present in the population and design optimal primers per SSR marker. Highly polymorphic markers can then be used to genotype other individuals from the same species.

Currently, there are a limited number of programs that can detect microsatellites directly from sequencing output, or that incorporates data cleaning and primer-designing processes in their workflows (Table 1). To perform microsatellite mining, these tools usually require either (1) a reference genome, what implies that they can be used only when the species of the study is well known or the analysis will need a previous hard work to get at least a decent draft of the species genome, or (2) they work with pre-processed long sequences (contigs) from individual sample libraries, thereby preventing the detection of highly polymorphic SSR loci. Also, they only consider non-enriched libraries what limitates their use in the recovery of polymorphic SSRs for individual identification^20^. Since most of the pipelines aim is not to find polymorphic SSRs, they do not make use of enriched libraries although they could perfectly process this type of data since the difference lies only in the proportion of sequences with repetitive patterns versus the total number of sequences. More recently, the MiMi^11^ pipeline has been developed to work directly from raw sequencing data of multiple individuals and, based on microsatellite prediction and calculation of allele frequencies, it can identify good priming regions. However, this pipeline requires quite a bit of data manipulation by the user, as quality trimming and microsatellite screening need to be performed externally (Palfinder Galaxy Service, https://palfinder.ls.manchester.ac.uk/) before importing the results into the MiMi pipeline for microsatellite selection and primer design. Moreover, to the best of our knowledge, none of these tools takes into consideration several important parameters in SSR identification and primer design from a singleplexed multi-individual library. The possibility of developing SSR loci from multiple samples in a single reaction is particularly desirable, as it reduces the overall costs of microsatellite development per species. Another concern about the existing tools is their dependence on external software and the need for the user to install all dependencies manually. A comprehensive description of the existing programs can be found in Table 1.

**Table 1:** Details on the existing integrative microsatellite development tools for SSR mining and primer design for direct comparison to micro-Primers.

	Micro-Primers	MiMi^#^	QDD	SSREnricher^#^	IDSSR^#^	FullSSR^#^	Krait	SSR Pipeline	GMATA^#^	CandiSSR^#^
	This study	Fox et al.^11^	Meglécz et al.^12^	Luo et al.^13^	Guang et al.^14^	Metz et al.^15^	Du et al.^16^	Miller et al.^17^	Wang et al.^18^	Xia et al*. *^19^
**Installation**										
	Auto-installation of all required software (through conda environment)	Software installation required (BioPython, MUSCLE, pandaseq)	Software installation required (RepeatMasker, NCBI nt database,bioperl, blast + , clustalW, primer3)	Software installation required ( Biopython, Perl, CD-HIT, muscle)	Software installation required (primer3)	Software installation required (primer3)	Software installation required (PySide2, pyfastx, numpy, requests, jinja2, appdirs, primer3-py, Cython, pyinstaller)	Software installation required (glibc)	Software installation required (e-PCR.exe)	Software installation required (Blast, Bioperl, MISA, ClustalW, Primer3)
**Library properties**										
DNA source	Multi-individual (uniplexed)	Multi-individual (multiplexed)	Single-, multi-individual	Single-, multi-individual	Single-, multi-individual	Single-individual	Single-individual	Single-individual	Single-, multi-individual	Multi-individual
Target fragments	Restriction fragments and shotgun	Shotgun fragments	Shotgun fragments	Assembled sequences	Shotgun fragments	Assembled sequences	Assembled sequences	Shotgun fragments	Assembled sequences	Shotgun fragments
**Data processing**										
Input files	Fastq (reads)	Fastq (reads)	Cleaned fastq/fasta (contigs or reads)	Fasta (contigs)	Fasta [reference genome] and cleaned fastq (reads)	Cleaned fasta (contigs)	Cleaned fasta (contigs)	Fastq and fasta	Cleaned fasta (contigs)	Cleaned fasta and reference
Adapter/quality filtering	Trimmomatic w/ Cutadapt	FastQC w/Trimmomatic (Palfinder Galaxy Service)	‒	‒	‒	‒	‒	SSR Pipeline	‒	‒
Paired read merge	FLASH	Pandaseq	‒	‒	‒	‒	‒	FLASH	‒	‒
SSR search	MISA w/ CD-HIT	Pal_finder (Palfinder Galaxy Service)	QDD	MISA w/ CD-HIT	IDSSR	FullSSR	Krait	SSR Pipeline	GMATA	MISA
Primer design	Primer3	Primer3 (Palfinder Galaxy Service)	Primer3	‒	Primer3 [reference]	Primer3	Primer3	‒	GMATA	Primer3
Loci filtering	Flanking length, repeat motif; observed and potential polymorphism, range of amplicon length	Repeat motif, observed polymorphism, SSR size-range	Minisatellite removal, flanking length, repeat motif, minimum coverage, locus specificity	Flanking length, repeat motif, observed polymorphism	Flanking length, minimum coverage, repeat motif, primer mismatch and specificity, observed polymorphism	Flanking length, repeat motif, imperfect repeat removal	Flanking length, compound, imperfect and perfect repeat motif, plots	Flanking length, repeat motif	Observed polymorphism, repeat motif, plots	Flanking length, loci coverage
Loci filtering (programs)	Micro-Primers	Pal_filter and PANDAseq* (Palfinder Galaxy Service) and MUSCLE (MiMi)	BLAST w/ RepeatMasker	SSREnricher	BLASTn; SOAP2 w/ SOAPindel	‒	‒	‒	‒	Blast, ClustalW
User interface	GUI	Galaxy (Palfinder only)	Galaxy	GUI	No	No	GUI	No	GUI	No

Information taken from the description of each software.

*This step is optional.

To tackle such limitations, we developed Micro-Primers, a tool that integrates a set of external programs into an automated pipeline, thereby allowing the perfect communication between programs through conditional formatting of input/output and reducing end-user data manipulation. Micro-Primers presents both a simple and intuitive GUI, with the basic parameters to be set and run in just a few clicks, and a command-line mode, where the user can manage the whole set of parameters or even switch on/off steps they do not want to be applied to their data. Overall, Micro-Primers represents a unique and easy framework for microsatellite development from multi-individual libraries (available at the GitHub repository https://github.com/FilAlves/micro-primers).

## Results

In this section, we show the experimental results obtained with our pipeline in a real case study. The analysis was then reproduced using the same data (adapted in some cases to adequate with the software requirements) in three pipelines from Table 1 capable of finding polymorphisms in the population dataset (MiMi, SSREnricher and GMATA).

### Micro-Primers’ output

**Figure 1:**
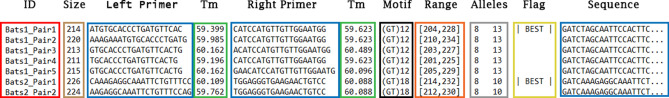
Micro-Primers’ output file capture. Columns show sequence ID, PCR amplicon length for the corresponding primer pair (size), sequence and melting temperature for left and right primer, SSR pattern (Motif), range of sizes for the SSR loci including all alleles (range), number of alleles found for the SSR and maximum number of alleles to expect from the difference between the longest and the shortest allele (Alleles), flag for best primer pair for the SSR loci (Flag) and the sequence from where the microsatellite was found and primers where designed from (Sequence).

The execution of Micro-Primers pipeline produces a single output file in plain text with useful information for the amplification of the SSR loci based on its representative sequence. Figure 1 shows a sample of file and how it is divided. It has twelve columns, and each line represents the primers designed by Primer3^21^ for each SSR recovered from the multi-individual sample. From left to right, the first column, in red, is the unique name of each cluster (ID) composed by a prefix (set by the user), the number of the loci and the primer pair number. Lines sharing the same loci number represent different primer pairs for the same SSR loci. The second column has the length (size) of the sequence resultant from PCR amplification using the respective primer pairs. The third and fourth columns are the forward primer sequence and its melting temperature. The fifth and sixth columns refer to the equivalent information for the reverse primer. In the seventh column, the specific motif found is shown with the number of repeats present in the representative sequence. The column identified as ‘Range’ shows the length span of the alleles detected for the same SSR. The nineth column contains the total number of alleles for the specific SSR loci. The tenth column indicates the potential number of alleles to be found in the population estimated from the difference between the longest and shortest alleles found. The eleventh column indicates the best combination of primer pairs for each loci (coded as “|BEST|”) as provided by Primer3 and the last column contain the representative sequence for the SSR loci from which the primers where designed.

### Micro-Primers performance analysis

The Micro-Primers pipeline was tested with a bat dataset from two different populations, Namibia and Botswana, with a total of 15 and 21 individuals respectively (the dataset is also available in the GitHub repository together with the Micro-Primers’ software). Samples were pooled, enriched for di- and tetra-repeat motifs separately, following a protocol modified from Garrett et al.^22^ and sequenced on an Illumina MiSeq v2 kit (250 cycles, paired-end).

The analysis was performed in a single core of an Intel i7 Octa-Core processor and 64 Gbytes of main memory and took 2 min for Micro-Primers to finish. It should be noted that the only processing step demanding high RAM memory is data trimming, carried out by the component; otherwise, minimal resources are needed. In addition, four different parameters configurations were tested to check the performance of the pipeline and evaluate the differences in the number of microsatellite loci detected. The maximum expected number of alleles to be found in each locus is 72 since the species is diploid. The pipeline’s execution was modified by changing the parameters at the GUI settings tab or modifying the Primer3 settings file, and the number of sequences remaining after each step is presented in Table 2. The four configurations tested were: (1) the default; (2) with activation of the ‘Special Search’ with a minimal difference between extreme alleles of 8; (3) with change of the flanking region length; and (4) modifying the difference in melting temperature between forward and reverse primers at Primer3 (MAX DIFF TM).

**Table 2:** Variation of the selected sequences during the steps of Micro-Primers.

MIN_ALLEL_CNT	5	5	5	5	5	5	5	5
SPECIAL DIF	0	1(8)	0	0	0	0	0	0
MIN FLANK LEN	50	50	25	75	100	50	50	50
MAX DIFF TM	0.5	0.5	0.5	0.5	0.5	0.2	1	2
Original FASTQ file				259,506				
Trimming				188,843				
Pair-end merge				130,603				
Filter 1				19,695				
Filter 2	8083	8083	9568	6616	3752	8083	8083	8083
Clusters	4924	4924	6049	3801	2453	4924	4924	4924
Filter 3	2092	2092	2110	2028	779	2092	2092	2092
Unique loci	26	104	28	23	20	26	26	26
Primers selected	23	83	23	20	15	25	21	21

As observed in Table 2, the numbers of sequences that comply the requirements in the first four pipeline steps are the same, since none of the tested configurations are applied at these levels. The pipeline output changes after Filter 2 depending on the configuration used. The implementation of the ‘Special Search’, based on the potential number of alleles per loci, shows substantial impact on the final number of loci kept and subsequent number of primers selected in comparison with the default setting based on the observed number of alleles. Specifically, when the ‘Special Search’ is activated and the minimal difference between the extreme alleles is set to 8, the number of SSR loci increases from 26 to 104, producing a total of 83 primer pairs. Also, variations in the minimal flanking region length at Filter 2 affect the number of sequences that will pass to the following steps, and consequently the number of SSR markers obtained at the end. Higher values in the flanking region parameter make the filter more restrictive, and fewer sequences will be clustered since they are not long enough and therefore shall not be kept. Importantly, there should be a compromise between the length of the flanking region and the capacity of Primer3 to design primers considering the parameter settings given. The shorter the flanking regions are, the more sequences will pass through the Filter 2, although most will not be processed by Primer3 since they will not have enough length for the primers to be designed without overlapping with the microsatellite region (this option can be activated in Micro-Primers to post filtering microsatellites with primers designed in the repeated pattern).

At the end of the pipeline, changes in the maximum difference of melting temperature between primers in Primer3 (MAX DIFF TM) induces variation in the number of primer pairs designed, as expected. Higher values in this parameter increase the capacity of Primer3 to find primer pairs in a sequence but, contrary to expectations, they may not necessarily be the most suitable and therefore fewer primers are selected at the end.

### Validation of Micro-Primers’ results

**Figure 2:**
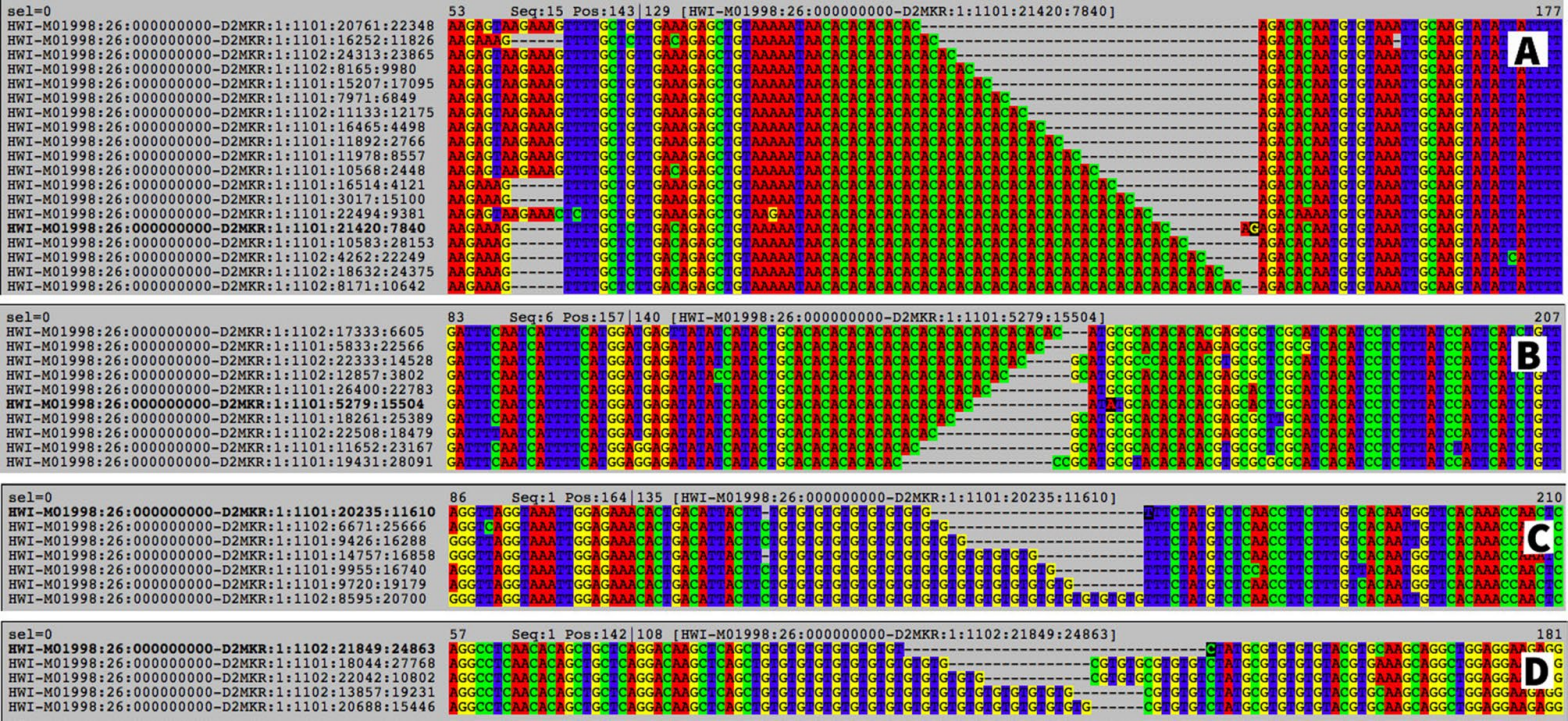
Visualization of the repeated region for the SSR loci Bats1 (**A**), Bats8 (**B**), Bats13 (**C**) and Bats20 (**D**) including the sequence ID of each of the alleles. SSR loci Bats1 was identified by Micro-Primers with 19 observed alleles and 19 expected, Bats8 loci was predicted with 10 observed alleles and 11 expected, Bats13 was predicted with 7 observed alleles and 13 expected, and Bats20 loci was predicted with 5 observed alleles and 12 expected.

Four random SSR loci out of the 23 found by Micro-Primers using the default configuration were validated by searching for each of the predicted alleles. Validation consists of retrieving from the original raw data (before any processing) the sequence including the repeated pattern to count the different alleles. The selected loci were Bats1, Bats8, Bats13 and Bats20 with 19, 10, 7 and 5 alleles, respectively. Alignments of the repeated region for the 4 SSR loci are shown in Fig. 2 together with the sequence ID of the sequences that have each allele. Full alignments in Phylip format are included as Supplementary material .

### Comparison of Micro-Primers with other pipelines

Micro-Primers performance was compared with three of the pipelines shown in Table 1 with similar functioning. The three chosen software were SSREnricher, GMATA and MiMi for including SSR polymorphism detection. SSREnricher and GMATA were fed with the pair reads trimmed and joint by FLASH^23^ in fasta format. Our efforts in comparing pipelines were hampered by data incompatibility:

The SSREnricher was not able to process the bat data despite the input file was in fasta format, demanding the input to come from Trinity software output. Trinity is an assembler, and the single action an assembler can conduct is to create consensus sequences, thereby reducing the number of microsatellite alleles. Since our sequence data was originated from enzyme digestion, independent fragments could not be assembled with other fragments.

The GMATA was executed with the clean joined pairs in a fasta format. The program run and managed to find microsatellites but the primer design could not be performed, and thus no plots were generated. Moreover, despite what is indicated in the program description, there is no function to perform the analysis of polymorphism. The pipeline originated a file with individual SSR prediction for each sequence and another with the overall statistics.

The MiMi was the only software that was able to run with the bat dataset (splitting the unique file into 36 single files, one for each individual). The pre-processing of the data prior to MiMi execution (trimming, adaptor removal and microsatellite detection) on the Galaxy server took more than 5 h and required a great amount of data manipulation by the user to upload the data, run each of the programs and download the results. Once the appropriate input was obtained, the MiMi program was able to process data in few seconds. It detected five microsatellite loci but all with low quality, thereby originating no results at the end. The five SSR loci detected were not comparable with the Micro-Primers results.

## Discussion

Nowadays, it can take up months for laboratories and researchers to analyze and select SSR loci and design primers for their projects. Usually, it requires the analysis of pre-processed sequencing data from individual samples in programs such as MISA^24^, and the manual selection of candidate loci for primer design in Primer3. The use of multi-individual assays reduces laboratory and sequencing costs, but it hampers data processing since results need to be compared to extract useful information and avoid replicated information across the samples, which likely explains why this procedure has not been fully embraced by researchers yet. Most selected SSR loci and subsequent PCR amplifications would likely be mediocre if manual SSR selection is applied, but also if clustering of flanking regions and the number of putative alleles is not considered, thereby generating markers with low variability across populations. The preparation of a microsatellite library from a single individual is easier and cheaper than from a multi-individual sample, particularly if using an enrichment step, and the posterior analysis deserves less analytical effort. However, processing a single individual hardly results on the detection of highly polymorphic loci in comparison to a multi-individual library, although the latter requires a more complex data handling.

These technical constraints could be easily overcome by the implementation of an automatic tool, allowing users to test distinct parameters and select the most suitable SSRs for their experiment in a few seconds. Towards this end, we have designed and implemented an automatic pipeline for microsatellite screening and primer design from multi-individual sequencing data, Micro-Primers, which can be applied on sequencing output from libraries produced by restriction enzyme digestion or from mechanical shearing experiments. There is a great advantage of implementing this tool due to its ability to surpass time-consuming steps such as the editing of input/output formats between successive programs, or the categorization and allele counting for every single SSR. First, all microsatellites individually identified need to be grouped by their ’true’ locus, considering that they are originated from different individuals and can be represented in multiple sequences. After the identification of all copies of the same microsatellite loci, the tool counts for the number of different unique alleles for the subsequent selection. This particular task can be very tedious if performed manually, as some of the locus can be represented by many elements.

Micro-Primers has a default configuration that has demonstrated to achieve a good balance between the number of loci and primer designed, however users should modify these parameters to adequate the results to their requirements. For example, the inclusion of the *SPECIAL SEARCH* option, which considers the maximum and minimum allele found in the SSR loci, incorporates in the decision-making the assumption about unsampled alleles for the species.

It is recommended that users scrutinize the final output of Micro-Primers prior to laboratorial processing. Despite the appropriate settings imposed, we observed various sources of variation associated with the different programs used, occasionally producing divergent or imprecise results. The observed bias on the final output is not related with the performance of Micro-Primers as it may also occur during manual processing. Specifically, (a) since most SSR sequences occur on the overlapping region, the software FLASH can produce some length variation while merging the paired-end reads by not recognizing the accurate position of the repeated pattern where sequences should be merged, thus creating ‘fake’ alleles; (b) based on the CD-HIT^25,26^ software, the creation of clusters based on the flanking regions can be arbitrary to some extent, due to the occurrence of amplification and sequencing errors that were not discarded at the first steps. Because of it, the group of sequences composing each cluster will depend on the seed sequence selected to originate each cluster; (c) the selection of the representative cluster for primer design is arbitrary. A cluster can be discarded whenever the representative does not produce suitable primer pairs in Primer3 (e.g., if the product size is out of the defined product range), however its effect was found to be marginal; and (d) Micro-Primers uses the starting position of the SSR loci provided by MISA to design primers in Primer3. However, in some situations, this position is incorrectly assigned (e.g., placed within the repetitive region), and thus unsuitable primer sets can be generated. We recommend users to always validate the primer sets given using the microsatellite sequence provided, or, whenever needed, to manually redesign primers by finding suitable binding positions in the sequence (e.g., using Primer3 online). Yet, only 2 out of the 23 results in the default configuration were affected by the creation of primers inside the repeated area itself. This can be avoid activating the option prior to run to eliminate results with any primer inside the SSR pattern but we would be losing good SSR loci that can be just solved re-designing a new primer pair. In case of libraries produced by restriction enzyme, like the one used in the experiment, the possibility of having errors of increasing or reducing the number of repeats at the merge step by FLASH is controlled by the selection of a proper restriction enzyme with a non-repeated pattern, so the SSR repeat will never be in the ends of the pair reads.

The use of Micro-Primers provides an unprecedented availability of candidate SSRs at a more reliable and faster pace than before, and retrieves relevant SSR information from multi-individual libraries not considered in other pipelines. The development of automatic pipelines is highly relevant for the scientific community since it can speed up the process and overcome the biases associated with the manual processing, while allowing the user to test various parameter choices by automatically running the process several times on the same dataset.

## Conclusions

We presented a novel, integrated and user-friendly pipeline for microsatellite searching and primer design from multi-individual sequencing data named Micro-Primers. Experimental results showed that Micro-Primers takes about 2 min to execute an entire analysis (300 k reads), from raw data to primer design, using just a single core of an Intel i7 Octa-Core processor with 64 Gbytes of main memory. The accuracy of Micro-Primers was tested identifying each of the predicted alleles for 4 SSR loci in the raw data prior to any processing.

To the best of our knowledge, this is the first pipeline to completely automatize the process for multi-individual libraries, allowing any researcher to make a fast characterization of a species prior to any extensive genotyping. It can be used through a GUI or command-line and, since it is implemented in Python, it is portable enough to be used in any kind of operating system, such as Windows, Linux or MacOs. Due to its simple design, it can be easily implemented in any type of computational language and library, or even incorporated in more complex software systems.

## Materials and methods

The main motivation of Micro-Primers is to eliminate issues regarding computational time and work, by performing an automated selection of candidate microsatellite loci and PCR primers. As such, several tools and scripts were integrated within Micro-Primers for discovering SSRs and designing the respective primers for further in vitro amplification. Micro-Primers has been installed and tested in Linux and MacOS. The installation can be done by cloning the software directly from Github or through conda installation where all the software versions and dependencies are installed automatically. Instructions are available at Micro-Primers Github page.

### Internal and external components

The Micro-Primers pipeline was written in Python version 3.6. The two main internal components were implemented using the following scripts: (1) install.py that includes all necessary pre-requisites for a proper installation of Micro-Primers, and (2) micro-primers.py, which is the main script that defined the pipeline. Analysis settings are described in the config.txt file, and parameters can be modified by the user accordingly to their own needs. The folder software, provided together with the Python scripts, holds all the scripts and external software employed by micro-primers.py.

The Micro-Primers pipeline integrates several external components, such as: (1) Trimmomatic^27^ for the removal of the sequencing adapters; (2) Cutadapt^28^ for the removal of the technology-specific adapter; (3) FLASH for the merging of paired-end reads (R1 and R2); (4) MISA for the SSR searching; (5) CD-HIT for the removal of redundancy; (6) Primer3 for primer design.

### Input files and pipeline

To run Micro-Primers, users only need to provide two FASTQ files corresponding to both ends of a paired-end sequencing run. The program can run as well in case of single-end sequencing by introducing the same file as forward and reverse since in the merge step they will be reduced to the original one. Samples should come from a library of pooled (untagged) individuals of the same species, so that the microsatellite selection can be optimized. SSR selection will be performed based on the number of alleles of each SSR loci, so the more heterogeneous the sample is (i.e., containing individuals from distinct populations across the species distribution), the better the outcome is expected to be. Reads can come from a microsatellite library built using a restriction enzyme and following an enrichment protocol such as the one described in^22^ or just from mechanical fragmentation methods. The enrichment protocol must be performed after digestion, so the target SSR motifs are the most represented strands in the final library. A fragment size selection is then performed on the enriched library to keep only fragments of an average length lower than the maximum sequencing length, to allow both paired-ends reads overlap when merged later on. The final fragment size is important for microsatellite screening and must comprise the full SSR pattern (variable in length) and the two flanking regions with fair length for primer design.

**Figure 3:**
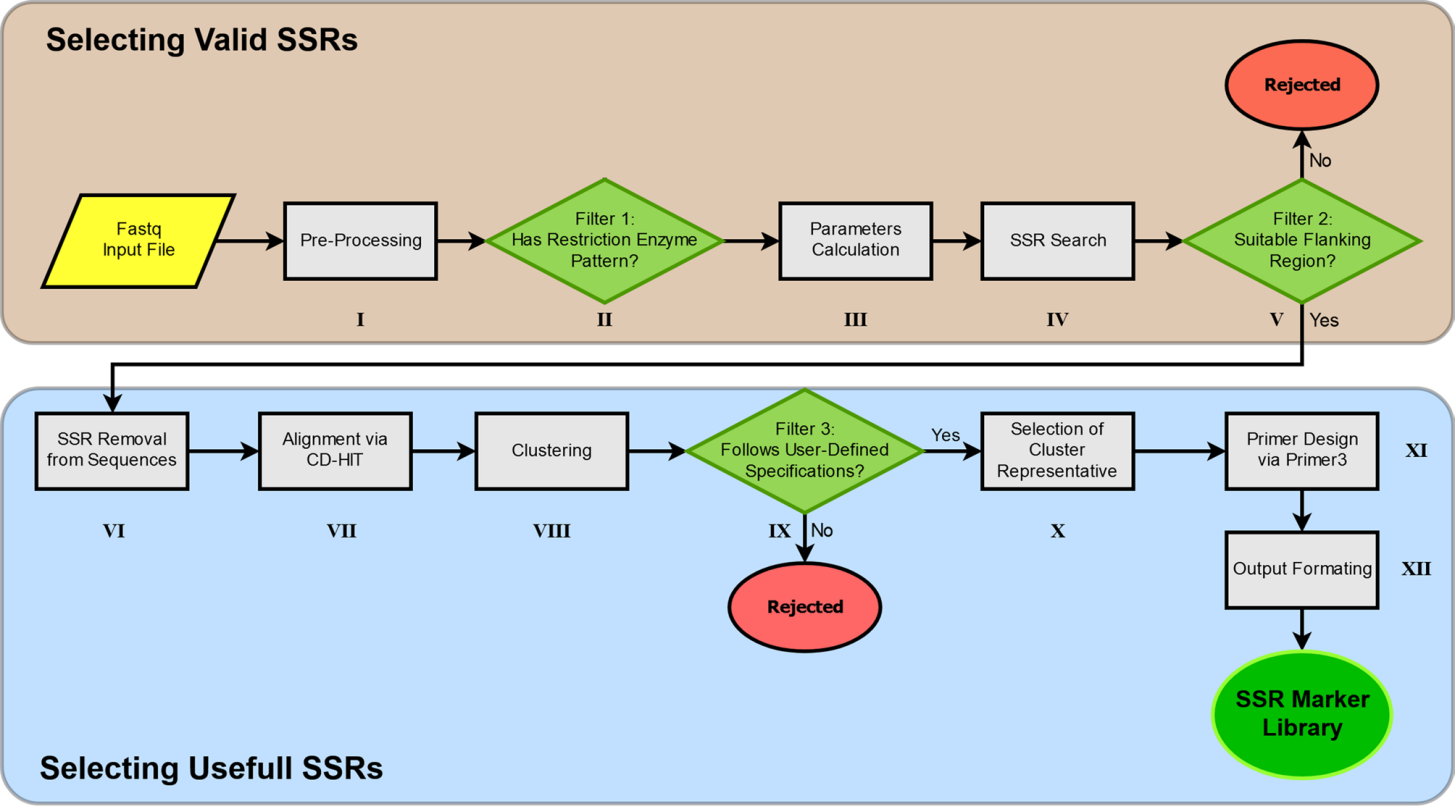
Flowchart of Micro-Primers. Green diamonds represent the different sequence filtering stages based on the parameters established by the user.

Additionally, prior to the execution of Micro-Primers, all the external components must be installed through the script install.py (only the first time). Once everything is set Micro-Primers can be launched executing the script micro-primers.py and the GUI will appear with the available options. For a complete list of parameters and to enter in command-line mode the user only needs to add the option “*-help*” to the micro-primers.py script. Upon execution, Micro-Primers will follow the flowchart described in Fig. 3. It begins using Trimmomatic and Cutadapt for the removal of sequencing and technology-specific adapters respectively, and both paired-end reads are merged via FLASH. When the input library is coming from digestion (and the option is not disabled) only sequence reads containing the restriction enzyme pattern are kept by the pipeline. Various parameters are then calculated and only the sequences that comply with the specifications of the users are selected. Next, the repeating region of SSRs is removed from sequences, and the flanking regions are aligned and assigned to a cluster using CD-HIT with the following parameters (− c = 0.90 − n = 10).

Sequences belonging to the same cluster are sorted and number of different alleles in the cluster are computed. Only clusters (i.e., SSR loci) with a minimal number of alleles (set by the user) are chosen, from which a random sequence among variants is selected as the representative of each locus. Every representative is then parsed into primer3 and, accordingly to the primer’s specifications set by the user, an output file will be generated that includes both primer information and number of alleles per SSR locus (see Fig. 1).

### Execution parameters

As described previously, the basic parameters required by Micro-Primers must be set in the different tabs of the program interface. In the main screen the input pair-end files and a prefix for the results should be indicated. The “Settings” button contains three sections with different parameters to be considered for the pipeline execution:

Section 1: Pre-processing: the user must indicate the sequence of the adapters used after the restriction enzyme action and the pattern remaining in the sequence after the digestion. If the data was not produced by restriction enzyme this option can be disabled at this section as well.

Section 2: Primers: here one or more SSR types can be excluded from the available options: c (compound), c* (compound with imperfection) and p1 to p6 (repeated motif of 1–6 nucleotides). A tuned Primer3 setting file can be selected in case the default one included in Micro-Primers is not convenient.

Section 3: Alleles: this section has important parameters to filter the SSR results produced by MISA software. Here we can find the “*Minimum number of alleles in cluster*” which indicates the minimum number of alleles for a SSR locus to be selected and it is based on the observed alleles (default value set to 5). In opposition, the parameter “*Minimum distance between alleles*” indicates the minimum potential number of alleles desired for each locus, assuming that not all alleles are represented in the multi-individual sample. Considering the difference between the alleles with higher and lower number of repeats, only loci that satisfy the minimum number of alleles indicated in this parameter are kept. This parameter is used only when the option “Special Search” is enabled (ON). Other parameters in this section are “*Minimum flank region length*” which will discard every sequence with at least one flanking region shorter than the indicated, and “*Minimum motif repetition*” that sets the minimum number of repetitions in a SSR to continue in the pipeline.

## Supplementary Information

Supplementary Information 1.

Supplementary Information 2.

Supplementary Information 3.

Supplementary Information 4.

## Data Availability

Data available at the GitHub repository https://github.com/FilAlves/micro-primers.
