# Supplementary material for: Automating microsatellite screening and primer design from multi-individual libraries using Micro-Primers
Source: Sci Rep. 2022 Jan 7;12:295. doi: 10.1038/s41598-021-04275-8 (PMC8741888; doi:10.1038/s41598-021-04275-8)
Supplement: Supplementary file 1 — Supplementary Information 1. [file 41598_2021_4275_MOESM1_ESM.pdf]

```
Alignment: /Mio/Papers/micro-primers/submission/verification/bats1_allele.phy
Seaview [blocks=10 fontsize=10 A4] on Sat Sep  4 15:21:48 2021

                                                    1
HWI-M01998:26:000000000-D2MKR:1:1101:20761:22348   GATCAGACTA CCGCTCATCA AGTATTCCTG
HWI-M01998:26:000000000-D2MKR:1:1101:16252:11826   GATCAGACTA CCGCTCATCA AGTATTCCTG
HWI-M01998:26:000000000-D2MKR:1:1102:24313:23865   GATCAGACTA CCGCTCATCA AGTATTCCTG
HWI-M01998:26:000000000-D2MKR:1:1102:8165:9980     GATCAGACTA CCGCTCATCA AGTATTCCTG
HWI-M01998:26:000000000-D2MKR:1:1101:15207:17095   GATCAGACTA CCGCTCACCA AGTATTCCTG
HWI-M01998:26:000000000-D2MKR:1:1101:7971:6849     GATCAGACTA CCGCTCATCA AGTATTCCTG
HWI-M01998:26:000000000-D2MKR:1:1102:11133:12175   GATCAGACTA CCGCTCATCA AGTATTCCTG
HWI-M01998:26:000000000-D2MKR:1:1101:16465:4498    GATCAGACTA CCGCTCATCA AGTATTCCTG
HWI-M01998:26:000000000-D2MKR:1:1101:11892:2766    GATCAGACTA CCGCTCATCA AGTATTCCTG
HWI-M01998:26:000000000-D2MKR:1:1101:11978:8557    GATCAGACTA CCGCTCATCA AGTATTCCTG
HWI-M01998:26:000000000-D2MKR:1:1101:10568:2448    GATCAGACTA CCGCTCATCA AGTATTCCTG
HWI-M01998:26:000000000-D2MKR:1:1101:16514:4121    GATCAGACTA CCGCTCATCA AGTATTCCTG
HWI-M01998:26:000000000-D2MKR:1:1101:3017:15100    GATCAGACTA CCGCTCATCA AGTATTCCTG
HWI-M01998:26:000000000-D2MKR:1:1101:22494:9381    GATCAGACTA CCGCTCATCA AGTATTCCTG
HWI-M01998:26:000000000-D2MKR:1:1101:21420:7840    GATCAGACTA CCGCTCATCA AGTATTCCTG
HWI-M01998:26:000000000-D2MKR:1:1101:10583:28153   ---------- ----GATCCC AAGCTTCCTG
HWI-M01998:26:000000000-D2MKR:1:1102:4262:22249    GATCAGACTA CCGCTCATCA AGTATTCCTG
HWI-M01998:26:000000000-D2MKR:1:1102:18632:24375   GATCAGACTA CCGCTCATCA AGTATTCCTG
HWI-M01998:26:000000000-D2MKR:1:1102:8171:10642    GATCAGACTA CCGCTCATCA AGTATTCCTG

                                                  31
HWI-M01998:26:000000000-D2MKR:1:1101:20761:22348   GAGGTCCTAC CATAGAGAAA GTAAGAGTAA
HWI-M01998:26:000000000-D2MKR:1:1101:16252:11826   GAGGTCCTAC CATAGAGAAA GTAAGAAAG-
HWI-M01998:26:000000000-D2MKR:1:1102:24313:23865   GAGGTCCTAC CATAGGGAAA GTAAGAGTAA
HWI-M01998:26:000000000-D2MKR:1:1102:8165:9980     GAGGTCCTAC CATAGAGAAA GTAAGAGTAA
HWI-M01998:26:000000000-D2MKR:1:1101:15207:17095   GAGGTCCTAC CATAGAGAAA GTAAGAGTAA
HWI-M01998:26:000000000-D2MKR:1:1101:7971:6849     GAGGTCCTAC CATAGGGAAA GTAAGAGTAA
HWI-M01998:26:000000000-D2MKR:1:1102:11133:12175   GAGGTCCTAC CATAGAGAAA GTAAGAGTAA
HWI-M01998:26:000000000-D2MKR:1:1101:16465:4498    GAGGTCCTAC CATAGGGAAA GTAAGAGTAA
HWI-M01998:26:000000000-D2MKR:1:1101:11892:2766    GAGGTCCTAC CATAGGGAAA GTAAGAGTAA
HWI-M01998:26:000000000-D2MKR:1:1101:11978:8557    GAGGTCCTAC CATAGGGAAA GTAAGAGTAA
HWI-M01998:26:000000000-D2MKR:1:1101:10568:2448    GAGGTTCTAC CATAGAGAAA GTAAGAGTAA
HWI-M01998:26:000000000-D2MKR:1:1101:16514:4121    GAGGTCCTAC CATAGAGAAA GTAAGAAAG-
HWI-M01998:26:000000000-D2MKR:1:1101:3017:15100    GAGGTCCTAC CATAGAGAAA GTAAGAAAG-
HWI-M01998:26:000000000-D2MKR:1:1101:22494:9381    GAGGTCCTAC CATAGAGAAA GTAAGAGTAA
HWI-M01998:26:000000000-D2MKR:1:1101:21420:7840    GAGGTCCTAC CATAGAGAAA GTAAGAAAG-
HWI-M01998:26:000000000-D2MKR:1:1101:10583:28153   GAGGTCCTAC CATAGAGAAA GTAAGAAAG-
HWI-M01998:26:000000000-D2MKR:1:1102:4262:22249    GAGGTCCTAC CATAGAGAAA GTAAGAAAG-
HWI-M01998:26:000000000-D2MKR:1:1102:18632:24375   GAGGTCCTAC CATAGAGAAA GTAAGAAAG-
HWI-M01998:26:000000000-D2MKR:1:1102:8171:10642    GAGGTCCTAC CATAGAGAAA GTAAGAAAG-

                                                  61
HWI-M01998:26:000000000-D2MKR:1:1101:20761:22348   GAAAGTTTTG CTGTTGAAAG AGCTGTAAAA
HWI-M01998:26:000000000-D2MKR:1:1101:16252:11826   -----TTTTG CTCTTGACAG AGCTGTAAAA
HWI-M01998:26:000000000-D2MKR:1:1102:24313:23865   GAAAGTTTTG CTGTTGAAAG AGCTGTAAAA
HWI-M01998:26:000000000-D2MKR:1:1102:8165:9980     GAAAGTTTTG CTGTTGAAAG AGCTGTAAAA
HWI-M01998:26:000000000-D2MKR:1:1101:15207:17095   GAAAGTTTTG CTGTTGAAAG AGCTGTAAAA
HWI-M01998:26:000000000-D2MKR:1:1101:7971:6849     GAAAGTTTTG CTGTTGAAAG AGCTGTAAAA
HWI-M01998:26:000000000-D2MKR:1:1102:11133:12175   GAAAGTTTTG CTGTTGAAAG AGCTGTAAAA
HWI-M01998:26:000000000-D2MKR:1:1101:16465:4498    GAAAGTTTTG CTGTTGAAAG AGCTGTAAAA
HWI-M01998:26:000000000-D2MKR:1:1101:11892:2766    GAAAGTTTTG CTGTTGAAAG AGCTGTAAAA
HWI-M01998:26:000000000-D2MKR:1:1101:11978:8557    GAAAGTTTTG CTGTTGAAAG AGCTGTAAAA
HWI-M01998:26:000000000-D2MKR:1:1101:10568:2448    GAAAGTTTTG CTGTTGACAG AGCTGTAAAA
HWI-M01998:26:000000000-D2MKR:1:1101:16514:4121    -----TTTTG CTGTTGAAAG AGCTGTAAAA
HWI-M01998:26:000000000-D2MKR:1:1101:3017:15100    -----TTTTG CTGTTGAAAG AGCTGTAAAA
HWI-M01998:26:000000000-D2MKR:1:1101:22494:9381    GAAACTCTTG CTGTTGAAAG AGCTGTAAGA
HWI-M01998:26:000000000-D2MKR:1:1101:21420:7840    -----TTTTG CTCTTGACAG AGCTGTAAAA
HWI-M01998:26:000000000-D2MKR:1:1101:10583:28153   -----TTTTG CTCTTGACAG AGCTGTAAAA
HWI-M01998:26:000000000-D2MKR:1:1102:4262:22249    -----TTTTG CTCTTGACAG AGCTGTAAAA
HWI-M01998:26:000000000-D2MKR:1:1102:18632:24375   -----TTTTG CTCTTGACAG AGCTGTAAAA
HWI-M01998:26:000000000-D2MKR:1:1102:8171:10642    -----TTTTG CTCTTGACAG AGCTGTAAAA
```

```
                                                       91
HWI-M01998:26:000000000-D2MKR:1:1101:20761:22348  ATAACACACA CACAC----- ----------
HWI-M01998:26:000000000-D2MKR:1:1101:16252:11826  ATAACACACA CACACAC--- ----------
HWI-M01998:26:000000000-D2MKR:1:1102:24313:23865  ATAACACACA CACACACAC- ----------
HWI-M01998:26:000000000-D2MKR:1:1102:8165:9980    ATAACACACA CACACACACA C---------
HWI-M01998:26:000000000-D2MKR:1:1101:15207:17095  ATAACACACA CACACACACA CAC-------
HWI-M01998:26:000000000-D2MKR:1:1101:7971:6849    ATAACACACA CACACACACA CACAC-----
HWI-M01998:26:000000000-D2MKR:1:1102:11133:12175  ATAACACACA CACACACACA CACACAC---
HWI-M01998:26:000000000-D2MKR:1:1101:16465:4498   ATAACACACA CACACACACA CACACACAC-
HWI-M01998:26:000000000-D2MKR:1:1101:11892:2766   ATAACACACA CACACACACA CACACACACA
HWI-M01998:26:000000000-D2MKR:1:1101:11978:8557   ATAACACACA CACACACACA CACACACACA
HWI-M01998:26:000000000-D2MKR:1:1101:10568:2448   ATAACACACA CACACACACA CACACACACA
HWI-M01998:26:000000000-D2MKR:1:1101:16514:4121   ATAACACACA CACACACACA CACACACACA
HWI-M01998:26:000000000-D2MKR:1:1101:3017:15100   ATAACACACA CACACACACA CACACACACA
HWI-M01998:26:000000000-D2MKR:1:1101:22494:9381   ATAACACACA CACACACACA CACACACACA
HWI-M01998:26:000000000-D2MKR:1:1101:21420:7840   ATAACACACA CACACACACA CACACACACA
HWI-M01998:26:000000000-D2MKR:1:1101:10583:28153  ATAACACACA CACACACACA CACACACACA
HWI-M01998:26:000000000-D2MKR:1:1102:4262:22249   ATAACACACA CACACACACA CACACACACA
HWI-M01998:26:000000000-D2MKR:1:1102:18632:24375  ATAACACACA CACACACACA CACACACACA
HWI-M01998:26:000000000-D2MKR:1:1102:8171:10642   ATAACACACA CACACACACA CACACACACA
```

```
                                                  121
HWI-M01998:26:000000000-D2MKR:1:1101:20761:22348  ---------- ---------- ---AGACACA
HWI-M01998:26:000000000-D2MKR:1:1101:16252:11826  ---------- ---------- ---AGACACA
HWI-M01998:26:000000000-D2MKR:1:1102:24313:23865  ---------- ---------- ---AGACACA
HWI-M01998:26:000000000-D2MKR:1:1102:8165:9980    ---------- ---------- ---AGACACA
HWI-M01998:26:000000000-D2MKR:1:1101:15207:17095  ---------- ---------- ---AGACACA
HWI-M01998:26:000000000-D2MKR:1:1101:7971:6849    ---------- ---------- ---AGACACA
HWI-M01998:26:000000000-D2MKR:1:1102:11133:12175  ---------- ---------- ---AGACACA
HWI-M01998:26:000000000-D2MKR:1:1101:16465:4498   ---------- ---------- ---AGACACA
HWI-M01998:26:000000000-D2MKR:1:1101:11892:2766   C--------- ---------- ---AGACACA
HWI-M01998:26:000000000-D2MKR:1:1101:11978:8557   CAC------- ---------- ---AGACACA
HWI-M01998:26:000000000-D2MKR:1:1101:10568:2448   CACAC----- ---------- ---AGACACA
HWI-M01998:26:000000000-D2MKR:1:1101:16514:4121   CACACAC--- ---------- ---AGACACA
HWI-M01998:26:000000000-D2MKR:1:1101:3017:15100   CACACACAC- ---------- ---AGACACA
HWI-M01998:26:000000000-D2MKR:1:1101:22494:9381   CACACACACA C--------- ---AGACAAA
HWI-M01998:26:000000000-D2MKR:1:1101:21420:7840   CACACACACA CAC------- -AGAGACACA
HWI-M01998:26:000000000-D2MKR:1:1101:10583:28153  CACACACACA CACAC----- ---AGACACA
HWI-M01998:26:000000000-D2MKR:1:1102:4262:22249   CACACACACA CACACAC--- ---AGACACA
HWI-M01998:26:000000000-D2MKR:1:1102:18632:24375  CACACACACA CACACACAC- ---AGACACA
HWI-M01998:26:000000000-D2MKR:1:1102:8171:10642   CACACACACA CACACACACA C--AGACACA
```

```
                                                  151
HWI-M01998:26:000000000-D2MKR:1:1101:20761:22348  ATGTGTAAAT TGCAAGTATA TTATTTTCTC
HWI-M01998:26:000000000-D2MKR:1:1101:16252:11826  ATGTGTAATT GCAAGTATAT TATTTTCTCT
HWI-M01998:26:000000000-D2MKR:1:1102:24313:23865  ATGTGTAAAT TGCAAGTATA TTATTTTCTC
HWI-M01998:26:000000000-D2MKR:1:1102:8165:9980    ATGTGTAAAT TGCAAGTATA TTATTTTCTC
HWI-M01998:26:000000000-D2MKR:1:1101:15207:17095  ATGTGTAAAT TGCAAGTATA TTATTTTCTC
HWI-M01998:26:000000000-D2MKR:1:1101:7971:6849    ATGTGTAAAT TGCAAGTATA TTATTTTCTC
HWI-M01998:26:000000000-D2MKR:1:1102:11133:12175  ATGTGTAAAT TGCAAGTATA TTATTTTCTC
HWI-M01998:26:000000000-D2MKR:1:1101:16465:4498   ATGTGTAAAT TGCAAGTATA TTATTTTCTC
HWI-M01998:26:000000000-D2MKR:1:1101:11892:2766   ATGTGTAAAT TGCAAGTATA TTATTTTCTC
HWI-M01998:26:000000000-D2MKR:1:1101:11978:8557   ATGTGTAAAT TGCAAGTATA TTATTTTCTC
HWI-M01998:26:000000000-D2MKR:1:1101:10568:2448   ATGTGTAAAT TGCAAGTATA TTATTTTCTC
HWI-M01998:26:000000000-D2MKR:1:1101:16514:4121   ATGTGTAAAT TGCAAGTATA TTATTTTCTC
HWI-M01998:26:000000000-D2MKR:1:1101:3017:15100   ATGTGTAAAT TGCAAGTATA TTATTTTCTC
HWI-M01998:26:000000000-D2MKR:1:1101:22494:9381   ATGTGTAAAT TGCAAGTATA TTATTTTCTC
HWI-M01998:26:000000000-D2MKR:1:1101:21420:7840   ATGTGTAAAT TGCAAGTATA TTATTTTCTC
HWI-M01998:26:000000000-D2MKR:1:1101:10583:28153  ATGTGTAAAT TGCAAGTATA TTATTTTCTC
HWI-M01998:26:000000000-D2MKR:1:1102:4262:22249   ATGTGTAAAT TGCAAGTATA TCATTTTCTC
HWI-M01998:26:000000000-D2MKR:1:1102:18632:24375  ATGTGTAAAT TGCAAGTATA TTATTTTCTC
HWI-M01998:26:000000000-D2MKR:1:1102:8171:10642   ATGTGTAAAT TGCAAGTATA TTATTTTCTC
```

```
                                             181
HWI-M01998:26:000000000-D2MKR:1:1101:20761:22348  TCTTAAGTAA TGTCTTTCTT AATAGCGTAG
HWI-M01998:26:000000000-D2MKR:1:1101:16252:11826  TTTAAGTAAT GTCTTTCTTA ATAGCGTAGC
HWI-M01998:26:000000000-D2MKR:1:1102:24313:23865  TCTTAAGTAA TGTCTTTCTT AATAGCGTAG
HWI-M01998:26:000000000-D2MKR:1:1102:8165:9980    CCTTAAGTAA TGTCTTTCTT AATAGCGTAG
HWI-M01998:26:000000000-D2MKR:1:1101:15207:17095  TCTTAAGTAA TGCCTTTCTT AATAGCGTAG
HWI-M01998:26:000000000-D2MKR:1:1101:7971:6849    TCTTAAGTAA TGTCTTTCTT AATAGAGTAG
HWI-M01998:26:000000000-D2MKR:1:1102:11133:12175  TCTTAAGTAA TGTCTTTCTT AATAGCGTAG
HWI-M01998:26:000000000-D2MKR:1:1101:16465:4498   TCTTAAGTAA TGTCTTTCTT AATAGCGTAG
HWI-M01998:26:000000000-D2MKR:1:1101:11892:2766   CCTTAAGTAA TGTCTTTCTT AATAGCGTAG
HWI-M01998:26:000000000-D2MKR:1:1101:11978:8557   TCTTAAGTAA TGTCTTTCTT AATAGCGTAG
HWI-M01998:26:000000000-D2MKR:1:1101:10568:2448   TCTTAAGTAA TGTCTTTCTT AATAGCGTAG
HWI-M01998:26:000000000-D2MKR:1:1101:16514:4121   TCTTAAGTAA TGTCTTTCTT AATAGCGTAG
HWI-M01998:26:000000000-D2MKR:1:1101:3017:15100   TCTTAAGTAA TGTCTTTCTT AATAGCGTAG
HWI-M01998:26:000000000-D2MKR:1:1101:22494:9381   TCTTAAGTAA TG----TCTT AATAGCGTAG
HWI-M01998:26:000000000-D2MKR:1:1101:21420:7840   TCTTAAGTAA TGTCTTTCTT AATAGCGTAG
HWI-M01998:26:000000000-D2MKR:1:1101:10583:28153  TCTTAAGTAA TGTCTTTCTT AATAGCGTAG
HWI-M01998:26:000000000-D2MKR:1:1102:4262:22249   TCTTAAGTAA TGTCTTTCTT AATAGCGTAG
HWI-M01998:26:000000000-D2MKR:1:1102:18632:24375  TCTTAAGTAA TGTCTTTCTT AATAGCGTAG
HWI-M01998:26:000000000-D2MKR:1:1102:8171:10642   TCTTAAGTAA TGTCTTTCTT AATAGCGTAG

                                             211
HWI-M01998:26:000000000-D2MKR:1:1101:20761:22348  CATTCAGAAT TATTCGAAAA GTTAATTGTA
HWI-M01998:26:000000000-D2MKR:1:1101:16252:11826  ATTCAGAATT ATTCGAAAAG TTAATTGTAG
HWI-M01998:26:000000000-D2MKR:1:1102:24313:23865  CATTCAGAAT TATTTGAAAA GTTAATTGTA
HWI-M01998:26:000000000-D2MKR:1:1102:8165:9980    CATTCAGAAT TATTTGAAAA GTTAATTGTA
HWI-M01998:26:000000000-D2MKR:1:1101:15207:17095  CATTCAGAAT TATTCGAAAA GTTAATTGTA
HWI-M01998:26:000000000-D2MKR:1:1101:7971:6849    CATTCAGAAT TATTCGAAAA GTTAATTGTA
HWI-M01998:26:000000000-D2MKR:1:1102:11133:12175  CATTCAGAAT TATTCCAAAA GTTAATCGTA
HWI-M01998:26:000000000-D2MKR:1:1101:16465:4498   CATTCAGAAT TATTTGAAAA GTTAATTGTA
HWI-M01998:26:000000000-D2MKR:1:1101:11892:2766   CATTCAGAAT TATTTGAAAA GTTAATTGTA
HWI-M01998:26:000000000-D2MKR:1:1101:11978:8557   CATTCAGAAT TATTTGAAAA GTTAATTGTA
HWI-M01998:26:000000000-D2MKR:1:1101:10568:2448   CATTCAGAAT TATTCGAAAA GTTAATTGTA
HWI-M01998:26:000000000-D2MKR:1:1101:16514:4121   CATTCAGAAT TATTCGGAAA GTTAATTGTA
HWI-M01998:26:000000000-D2MKR:1:1101:3017:15100   CATTCAGAAT TATTCGGAAA GTTAATTGTA
HWI-M01998:26:000000000-D2MKR:1:1101:22494:9381   CATTCAGAAT TATTCGAAAA GTTAATTGTA
HWI-M01998:26:000000000-D2MKR:1:1101:21420:7840   CATTCAGAAT TATTCGAAAA GTTAATTGTA
HWI-M01998:26:000000000-D2MKR:1:1101:10583:28153  CATTCAGAAT TATTCGAAAA GTTAATTGTA
HWI-M01998:26:000000000-D2MKR:1:1102:4262:22249   CATTCAGAAT TATTCGAAAA GTTAATTGTA
HWI-M01998:26:000000000-D2MKR:1:1102:18632:24375  CATTCAGAAT TATTCGAAAA GTTAATTGTA
HWI-M01998:26:000000000-D2MKR:1:1102:8171:10642   CATTCAGAAT TATTCGAAAA GTTAATTGTA

                                             241
HWI-M01998:26:000000000-D2MKR:1:1101:20761:22348  GGTCATGGAA AATCTGAGAG AGTATTGTGT
HWI-M01998:26:000000000-D2MKR:1:1101:16252:11826  GTCATGGAAA ATCTGAGAGA GTGTTGTGTA
HWI-M01998:26:000000000-D2MKR:1:1102:24313:23865  GGTCATGGAA AATCCGAGAG AGTATTGTGT
HWI-M01998:26:000000000-D2MKR:1:1102:8165:9980    GGTCATGGAA AATCTGAGAG AGTATTGTGT
HWI-M01998:26:000000000-D2MKR:1:1101:15207:17095  GGTCATGGAA AATCTGAGAG AGTATTGTGT
HWI-M01998:26:000000000-D2MKR:1:1101:7971:6849    GGTCATGGAA AATCTGAGAG AGTATTGTGT
HWI-M01998:26:000000000-D2MKR:1:1102:11133:12175  GGTCATGGAA AATCTGAGAG AGTATTGTGT
HWI-M01998:26:000000000-D2MKR:1:1101:16465:4498   GGTCATGGAA AATCTGAGAG AGTATTGTGT
HWI-M01998:26:000000000-D2MKR:1:1101:11892:2766   GGTCATGGAA AATCTGAGAG AGTATTGTGT
HWI-M01998:26:000000000-D2MKR:1:1101:11978:8557   GGTCATGGAA AATCTGAGAG AGTATTGTGT
HWI-M01998:26:000000000-D2MKR:1:1101:10568:2448   GGTCATGGAA AATCTGAGAG AGTATTGTGT
HWI-M01998:26:000000000-D2MKR:1:1101:16514:4121   GGTCATGGAA AATCTGAGAG AGTATTGTGT
HWI-M01998:26:000000000-D2MKR:1:1101:3017:15100   GGTCATGGAA AATCTGAGAG AGTATTGTGT
HWI-M01998:26:000000000-D2MKR:1:1101:22494:9381   GGTCATGGAA AATCTGAGAG AGTATTGTGT
HWI-M01998:26:000000000-D2MKR:1:1101:21420:7840   GGTCATGGAA AATCTGAGAG AGTGTTGTGT
HWI-M01998:26:000000000-D2MKR:1:1101:10583:28153  GGTCATGGAA AATCTGCGAG AGTATTGTGT
HWI-M01998:26:000000000-D2MKR:1:1102:4262:22249   GGTCATGGAA AATCTGAGAG AGTATTGTGT
HWI-M01998:26:000000000-D2MKR:1:1102:18632:24375  GGTCATGGAA AATCTGAGAG AGTATTGTGT
HWI-M01998:26:000000000-D2MKR:1:1102:8171:10642   GGTCATGGAA AATCTGAGAG AGTATTGTGT
```

```
                                                    271
HWI-M01998:26:000000000-D2MKR:1:1101:20761:22348  AAAGAGAAAT TCATTTATCT AGTCACTACC
HWI-M01998:26:000000000-D2MKR:1:1101:16252:11826  AAGAGAAATT CATTTATCTA GTCACTACCC
HWI-M01998:26:000000000-D2MKR:1:1102:24313:23865  AAAGAGAAAT TCATTTATCT AGTCACTACC
HWI-M01998:26:000000000-D2MKR:1:1102:8165:9980    AAAGAGAAAT TCATTTATCT AGTCACTACC
HWI-M01998:26:000000000-D2MKR:1:1101:15207:17095  AAAGAGAAAT TCATTTATCT AGTCACTACC
HWI-M01998:26:000000000-D2MKR:1:1101:7971:6849    AAAGAGAAAT TCATTTATCT AGTCACTACC
HWI-M01998:26:000000000-D2MKR:1:1102:11133:12175  AAAGAGAAAT TCATTTATCT AGCCACTACC
HWI-M01998:26:000000000-D2MKR:1:1101:16465:4498   AAAGAGAAAT TCATTTATCT AGTCACTACC
HWI-M01998:26:000000000-D2MKR:1:1101:11892:2766   AAAGAGAAAT TCATTTATCT AGTCACTACC
HWI-M01998:26:000000000-D2MKR:1:1101:11978:8557   AAAGAGAAAT TCATTTATCT AGTCACTACC
HWI-M01998:26:000000000-D2MKR:1:1101:10568:2448   AAAGAGAAAT TCATTTATCT AGTCACTACC
HWI-M01998:26:000000000-D2MKR:1:1101:16514:4121   AAAGAGAAAT TCATTTATCT AGTCACTACC
HWI-M01998:26:000000000-D2MKR:1:1101:3017:15100   AAAGAGAAAT TCATTTATCT AGTCACTACC
HWI-M01998:26:000000000-D2MKR:1:1101:22494:9381   AAAGAGAAAT TCATTTATCT AGTCACTACC
HWI-M01998:26:000000000-D2MKR:1:1101:21420:7840   AAAGAGAAAT TCATTTATCT AGTCACTACC
HWI-M01998:26:000000000-D2MKR:1:1101:10583:28153  AAAGAGAAAT TCATTTATCT AGTCACTACC
HWI-M01998:26:000000000-D2MKR:1:1102:4262:22249   AAAGAGAAAT TCATTTATCT AGTCACTACC
HWI-M01998:26:000000000-D2MKR:1:1102:18632:24375  AAAGAGAAAT TCATTTATCT AGTCACTACC
HWI-M01998:26:000000000-D2MKR:1:1102:8171:10642   AAAGAGAAAT TCATTTATCT AGTCACTACC

                                                    301
HWI-M01998:26:000000000-D2MKR:1:1101:20761:22348  CCTAAGATC
HWI-M01998:26:000000000-D2MKR:1:1101:16252:11826  CTAAGATC
HWI-M01998:26:000000000-D2MKR:1:1102:24313:23865  CCTAAGATC
HWI-M01998:26:000000000-D2MKR:1:1102:8165:9980    CCTAAGATC
HWI-M01998:26:000000000-D2MKR:1:1101:15207:17095  CCTAAGATC
HWI-M01998:26:000000000-D2MKR:1:1101:7971:6849    CCTAAGATC
HWI-M01998:26:000000000-D2MKR:1:1102:11133:12175  CCAAAGATC
HWI-M01998:26:000000000-D2MKR:1:1101:16465:4498   CCTAAGATC
HWI-M01998:26:000000000-D2MKR:1:1101:11892:2766   CCTAAGATC
HWI-M01998:26:000000000-D2MKR:1:1101:11978:8557   CCTAAGATC
HWI-M01998:26:000000000-D2MKR:1:1101:10568:2448   CCTAAGATC
HWI-M01998:26:000000000-D2MKR:1:1101:16514:4121   CCTAAGATC
HWI-M01998:26:000000000-D2MKR:1:1101:3017:15100   CCTAAGATC
HWI-M01998:26:000000000-D2MKR:1:1101:22494:9381   CCTAAGATC
HWI-M01998:26:000000000-D2MKR:1:1101:21420:7840   CCTAAGATC
HWI-M01998:26:000000000-D2MKR:1:1101:10583:28153  CCTAAGATC
HWI-M01998:26:000000000-D2MKR:1:1102:4262:22249   CCTAAGATC
HWI-M01998:26:000000000-D2MKR:1:1102:18632:24375  CCTAAGATC
HWI-M01998:26:000000000-D2MKR:1:1102:8171:10642   CCTAAGATC
```
