# Supplementary material for: Automating microsatellite screening and primer design from multi-individual libraries using Micro-Primers
Source: Sci Rep. 2022 Jan 7;12:295. doi: 10.1038/s41598-021-04275-8 (PMC8741888; doi:10.1038/s41598-021-04275-8)
Supplement: Supplementary file 2 — Supplementary Information 2. [file 41598_2021_4275_MOESM2_ESM.pdf]

```
Alignment: /Mio/Papers/micro-primers/submission/verification/bats13_allele.phy
Seaview [blocks=10 fontsize=10 A4] on Mon Aug 30 16:50:59 2021

                                           1
HWI-M01998:26:000000000-D2MKR:1:1101:20235:11610  GATCTCCTT- ---TCATCAT AACTTTGAGT
HWI-M01998:26:000000000-D2MKR:1:1102:6671:25666   GATCTCCTCT CATCATAACT TTATTTGAGT
HWI-M01998:26:000000000-D2MKR:1:1101:9426:16288   GATCTCCTT- ---TCATCAT AACTTTGAGT
HWI-M01998:26:000000000-D2MKR:1:1101:14757:16858  GATCTCCTTT CATCATAACT TTATTTGAGT
HWI-M01998:26:000000000-D2MKR:1:1101:9955:16740   GATCTCCTT- ---TCATCAT AACTTTGAGT
HWI-M01998:26:000000000-D2MKR:1:1101:9720:19179   GATCTCCTT- ---TCATCAT AACTTTGAGT
HWI-M01998:26:000000000-D2MKR:1:1102:8595:20700   GATCTCCTTT CATCTTAACT TTATTTGAGT

                                          31
HWI-M01998:26:000000000-D2MKR:1:1101:20235:11610  AACTTATTGT TAAATCCGTA GTTCACACTC
HWI-M01998:26:000000000-D2MKR:1:1102:6671:25666   AACTTATTGT TAAATCTGTA GTTCACATTC
HWI-M01998:26:000000000-D2MKR:1:1101:9426:16288   AACTTATTGT TAAGTCCGTA GTTCACATTC
HWI-M01998:26:000000000-D2MKR:1:1101:14757:16858  AACTTATTGT TAAATCCGTA GTTCACATTC
HWI-M01998:26:000000000-D2MKR:1:1101:9955:16740   AACTTATTGT TAAATCCGTA GTTCACACTC
HWI-M01998:26:000000000-D2MKR:1:1101:9720:19179   AACTTATTGT TAAATCCGTA GTTCACACTC
HWI-M01998:26:000000000-D2MKR:1:1102:8595:20700   AACTTATTGT TAAATCCGTA GTTCACATTC

                                          61
HWI-M01998:26:000000000-D2MKR:1:1101:20235:11610  ACATCAGGGT ACAGGAAGGT GTGAGAGGTT
HWI-M01998:26:000000000-D2MKR:1:1102:6671:25666   ACATCAGGGT ACAGGAAGCT CTGAGAGGTC
HWI-M01998:26:000000000-D2MKR:1:1101:9426:16288   ACATCAGGGT ACAGGAAGTT CTGAGGGGTT
HWI-M01998:26:000000000-D2MKR:1:1101:14757:16858  ACATCAGGGT ACAGGAAGGT CTGAGGGGTT
HWI-M01998:26:000000000-D2MKR:1:1101:9955:16740   ACATCAGGGT ACAGGAAGTT CTGAGAGGTT
HWI-M01998:26:000000000-D2MKR:1:1101:9720:19179   ACATCAGGGT ACAGGAAGTT CTGAGAGGTT
HWI-M01998:26:000000000-D2MKR:1:1102:8595:20700   ACATCAGGGT ACAGGAAGGT GTGAGGGGTT

                                          91
HWI-M01998:26:000000000-D2MKR:1:1101:20235:11610  AGGTAAATTG GAGAAACACT GACATTACTT
HWI-M01998:26:000000000-D2MKR:1:1102:6671:25666   AGGTAAATTG GAGAAACACT GACATTACTT
HWI-M01998:26:000000000-D2MKR:1:1101:9426:16288   AGGTAAATTG GAGAAACACT GACATTACTT
HWI-M01998:26:000000000-D2MKR:1:1101:14757:16858  AGGTAAATTG GAGAAACACT GACATTACTT
HWI-M01998:26:000000000-D2MKR:1:1101:9955:16740   AGGTAAATTG GAGAAACACT GACATTACTT
HWI-M01998:26:000000000-D2MKR:1:1101:9720:19179   AGGTAAATTG GAGAAACACT GACATTACTT
HWI-M01998:26:000000000-D2MKR:1:1102:8595:20700   AGGTAAATTG GAGAAACACT GACATTACTT

                                         121
HWI-M01998:26:000000000-D2MKR:1:1101:20235:11610  -TGTGTGTGT GTGTGTGTG- ----------
HWI-M01998:26:000000000-D2MKR:1:1102:6671:25666   CTGTGTGTGT GTGTGTGTGT G---------
HWI-M01998:26:000000000-D2MKR:1:1101:9426:16288   CTGTGTGTGT GTGTGTGTGT GTG-------
HWI-M01998:26:000000000-D2MKR:1:1101:14757:16858  -TGTGTGTGT GTGTGTGTGT GTGTGTGTGT
HWI-M01998:26:000000000-D2MKR:1:1101:9955:16740   CTGTGTGTGT GTGTGTGTGT GTGTGTGTGT
HWI-M01998:26:000000000-D2MKR:1:1101:9720:19179   CTGTGTGTGT GTGTGTGTGT GTGTGTGTGT
HWI-M01998:26:000000000-D2MKR:1:1102:8595:20700   CTGTGTGTGT GTGTGTGTGT GTGTGTGTGT

                                         151
HWI-M01998:26:000000000-D2MKR:1:1101:20235:11610  ---------- ---TTTCTAT GTCTCAACCT
HWI-M01998:26:000000000-D2MKR:1:1102:6671:25666   ---------- ---TTTCTAT GTCTCAACCT
HWI-M01998:26:000000000-D2MKR:1:1101:9426:16288   ---------- ---TTTCTAT GTCTCAACCT
HWI-M01998:26:000000000-D2MKR:1:1101:14757:16858  G--------- ---TTTCTAT GTCTCAACCT
HWI-M01998:26:000000000-D2MKR:1:1101:9955:16740   GTG------- ---TTTCTAT GTCTCCACCT
HWI-M01998:26:000000000-D2MKR:1:1101:9720:19179   GTGTG----- ---TTTCTAT GTCTCAACCT
HWI-M01998:26:000000000-D2MKR:1:1102:8595:20700   GTGTGTGTGT GTGTTTCTAT GTCTCAACCT

                                         181
HWI-M01998:26:000000000-D2MKR:1:1101:20235:11610  TCTTTGTCAC AATGGTTCAC AAACCAACTC
HWI-M01998:26:000000000-D2MKR:1:1102:6671:25666   TCTTTGTCAC AATTGTTCAC AAACCAACTC
HWI-M01998:26:000000000-D2MKR:1:1101:9426:16288   TCTTTGTCAC AATTGTTCAC AAACCAACTC
HWI-M01998:26:000000000-D2MKR:1:1101:14757:16858  TCTTTGTCAC AATGGTTCAC AAACCAAATC
HWI-M01998:26:000000000-D2MKR:1:1101:9955:16740   TCTTTGTTAC AATGGTTCAC AAACCAACTC
HWI-M01998:26:000000000-D2MKR:1:1101:9720:19179   TCTTTGTCAC AATGGTTCAC AAACCAACTC
HWI-M01998:26:000000000-D2MKR:1:1102:8595:20700   TCTTTGTCAC AATTGTTCAC AAACCAACTC

                                         211
HWI-M01998:26:000000000-D2MKR:1:1101:20235:11610  ATGCTGCTTT CCTTTCAGTT CACAAAGCAG
HWI-M01998:26:000000000-D2MKR:1:1102:6671:25666   ATGCAGCTTT CCATTCAGTT CACAAAGCAG
HWI-M01998:26:000000000-D2MKR:1:1101:9426:16288   ATGCAGCTTT CCATTCAGTT CACAAAGCAG
HWI-M01998:26:000000000-D2MKR:1:1101:14757:16858  ATGCAGCTTT CCATTCAGTT CACAAAGCAG
HWI-M01998:26:000000000-D2MKR:1:1101:9955:16740   ATGCAGCTTT CCATTCAGTT CACAAAGCAG
HWI-M01998:26:000000000-D2MKR:1:1101:9720:19179   ATGCAGCTTT TCATTCAGTT CACAAAGCAG
HWI-M01998:26:000000000-D2MKR:1:1102:8595:20700   ATGCATCTTT CCATTCAGTT CACAAAGCAG
```

```
                                                     241
HWI-M01998:26:000000000-D2MKR:1:1101:20235:11610  CTGGACTTCC AGGTCCCGGG CTTGACCATC
HWI-M01998:26:000000000-D2MKR:1:1102:6671:25666   CTGTTCTTCC AGGTCCCGGG CTTGACCATC
HWI-M01998:26:000000000-D2MKR:1:1101:9426:16288   CTGGACTTCC AGGTCCCGGG CTTGACCATC
HWI-M01998:26:000000000-D2MKR:1:1101:14757:16858  CTGGACTTCC AGGTCCCGGG CTTGACCATC
HWI-M01998:26:000000000-D2MKR:1:1101:9955:16740   CTGGACTTCC AGGTCCCGGG CTTGACCATC
HWI-M01998:26:000000000-D2MKR:1:1101:9720:19179   CTGGACTTCC AGGTCCCGGG CTTGACCATC
HWI-M01998:26:000000000-D2MKR:1:1102:8595:20700   CTGGACTTCC AGGTCCCGGG CTTGACCATC

                                                     271
HWI-M01998:26:000000000-D2MKR:1:1101:20235:11610  CCAGGATAGG AATGAATTCA GCAGGATTGC
HWI-M01998:26:000000000-D2MKR:1:1102:6671:25666   CCAGGATAGG AATGAATTCA GCAGGATTGC
HWI-M01998:26:000000000-D2MKR:1:1101:9426:16288   CCAGGATAGG AATGAATTCA GCAGGATTGT
HWI-M01998:26:000000000-D2MKR:1:1101:14757:16858  CCAGGATAGG AATGAATTCA GCAGGATTGC
HWI-M01998:26:000000000-D2MKR:1:1101:9955:16740   CCAGGATAGG AATGAATTCA GCAGGATTGC
HWI-M01998:26:000000000-D2MKR:1:1101:9720:19179   CCAGGATAGG AATGAATTTA GCAGGATTGC
HWI-M01998:26:000000000-D2MKR:1:1102:8595:20700   CCAGGATAGG AATGAATTCA GTAGGATTGC

                                                     301
HWI-M01998:26:000000000-D2MKR:1:1101:20235:11610  CACCAGGGGA TGGAACATTC TAGAAAGCGT
HWI-M01998:26:000000000-D2MKR:1:1102:6671:25666   CACCAGGGGA TGGAACATTC CAGAAAGCGT
HWI-M01998:26:000000000-D2MKR:1:1101:9426:16288   CATCAGGGGA TGGAACATTC TAGAAAGCGT
HWI-M01998:26:000000000-D2MKR:1:1101:14757:16858  CACCAGGGGA TGGAACATTC TAGAAAGCGT
HWI-M01998:26:000000000-D2MKR:1:1101:9955:16740   CACCAGGGGA TGGAACATTC TAGAAAGCGT
HWI-M01998:26:000000000-D2MKR:1:1101:9720:19179   CACCAGGGGA TGGAACATTC TAGAAAGCGT
HWI-M01998:26:000000000-D2MKR:1:1102:8595:20700   CACCAGGGGA TGGGACATTC TAGAAAGCGT

                                                     331
HWI-M01998:26:000000000-D2MKR:1:1101:20235:11610  TGGCCTATAC AATGGAATCC ATACACCTCT
HWI-M01998:26:000000000-D2MKR:1:1102:6671:25666   TGGCCTATAC AATGGAATCC ATACACCTCT
HWI-M01998:26:000000000-D2MKR:1:1101:9426:16288   TGGCCTATAC AATGGAATCC ATATACCTCT
HWI-M01998:26:000000000-D2MKR:1:1101:14757:16858  TGGCCTATAC AATGGAATCC ATACACCTCT
HWI-M01998:26:000000000-D2MKR:1:1101:9955:16740   TGGCCTATAC AATGGAATCC ATACACCTCT
HWI-M01998:26:000000000-D2MKR:1:1101:9720:19179   TGGCCTATAC AATGGAATCC ATACACCTCT
HWI-M01998:26:000000000-D2MKR:1:1102:8595:20700   TGGCCTATAC AATGCAATCC ATACACCTCT

                                                     361
HWI-M01998:26:000000000-D2MKR:1:1101:20235:11610  CTCGCTATCC CCAAGTCTGC TACCCTTGGC
HWI-M01998:26:000000000-D2MKR:1:1102:6671:25666   CTCGCTATCC CCAAGTCTCC TACTCTTGGC
HWI-M01998:26:000000000-D2MKR:1:1101:9426:16288   CTCGCTATCC CCAAGTCTCC TACCCTTGGC
HWI-M01998:26:000000000-D2MKR:1:1101:14757:16858  CTCGCTATCC CCAAGTCTCC TACCCTTGGC
HWI-M01998:26:000000000-D2MKR:1:1101:9955:16740   CTCGCTATCC CCAAGTCTCC TACCCTTGGC
HWI-M01998:26:000000000-D2MKR:1:1101:9720:19179   CTCGCTATCC CCAAGTCTCC TACCCTTGGC
HWI-M01998:26:000000000-D2MKR:1:1102:8595:20700   CTCGCTATCC CCAAGTCTCC AACCCTTGGC

                                                     391
HWI-M01998:26:000000000-D2MKR:1:1101:20235:11610  AAAATCCCCT AATTGACTTT CACACCTGCA
HWI-M01998:26:000000000-D2MKR:1:1102:6671:25666   AAAATCCCCT AATAGACTTT CACACCTGCA
HWI-M01998:26:000000000-D2MKR:1:1101:9426:16288   AAAATCCCCT AATTGATTTT CACACCTGCA
HWI-M01998:26:000000000-D2MKR:1:1101:14757:16858  AAAATCCCCT AATTGACTTT CACACCTGCA
HWI-M01998:26:000000000-D2MKR:1:1101:9955:16740   AAAATCCCCT AATTGACTTT CACACCTGCA
HWI-M01998:26:000000000-D2MKR:1:1101:9720:19179   AAAATCCCCT AATTGACTTT CACACCTGCA
HWI-M01998:26:000000000-D2MKR:1:1102:8595:20700   AAAATCCCCT AATTGACTTT CACACCTGCA

                                                     421
HWI-M01998:26:000000000-D2MKR:1:1101:20235:11610  AGTCTGACTC TGATC
HWI-M01998:26:000000000-D2MKR:1:1102:6671:25666   AGTCTGACTC TGATC
HWI-M01998:26:000000000-D2MKR:1:1101:9426:16288   AGTCTGACTC TGATC
HWI-M01998:26:000000000-D2MKR:1:1101:14757:16858  AGTCTGACTC TGATC
HWI-M01998:26:000000000-D2MKR:1:1101:9955:16740   AGTCTGACTC TGATC
HWI-M01998:26:000000000-D2MKR:1:1101:9720:19179   AGTCTGACTC TGATC
HWI-M01998:26:000000000-D2MKR:1:1102:8595:20700   AGTCTGACTC TGATC
```
