# Supplementary material for: Automating microsatellite screening and primer design from multi-individual libraries using Micro-Primers
Source: Sci Rep. 2022 Jan 7;12:295. doi: 10.1038/s41598-021-04275-8 (PMC8741888; doi:10.1038/s41598-021-04275-8)
Supplement: Supplementary file 3 — Supplementary Information 3. [file 41598_2021_4275_MOESM3_ESM.pdf]

```
Alignment: /Mio/Papers/micro-primers/submission/verification/bats20_allele.fasta
Seaview [blocks=10 fontsize=10 A4] on Sat Sep  4 15:35:00 2021

                                                  1
HWI-M01998:26:000000000-D2MKR:1:1102:21849:24863  GATCTATCCA CGACTGTCAC CACGTTTCAA
HWI-M01998:26:000000000-D2MKR:1:1101:18044:27768  GATCTATCCA CGACTGTCAC CACGTTTCAA
HWI-M01998:26:000000000-D2MKR:1:1102:22042:10802  GATCTATCCA CGACTGTCAC CACGTTTCAA
HWI-M01998:26:000000000-D2MKR:1:1102:13857:19231  GATCTATCCA CGACTGTCAC CACGTTTCAA
HWI-M01998:26:000000000-D2MKR:1:1101:20688:15446  GATCTATCCA CGACTGTCAC CACGTTTCAA

                                                 31
HWI-M01998:26:000000000-D2MKR:1:1102:21849:24863  TGACCTGAGG GGACTGGACC TGAGCTAGGC
HWI-M01998:26:000000000-D2MKR:1:1101:18044:27768  TGACCTGAGG GGACTGGACC TGAGCTAGGC
HWI-M01998:26:000000000-D2MKR:1:1102:22042:10802  TGACCTGAGG GGACTGGACC TGAGCTAGGC
HWI-M01998:26:000000000-D2MKR:1:1102:13857:19231  TGACCTGAGG GGACTGGACC TGAGCTAGGC
HWI-M01998:26:000000000-D2MKR:1:1101:20688:15446  TGACCTGAGG GGACTGGACC TGAGCTAGGC

                                                 61
HWI-M01998:26:000000000-D2MKR:1:1102:21849:24863  CTCAACACAG CTGCTCAGGA CAAGCTCAGC
HWI-M01998:26:000000000-D2MKR:1:1101:18044:27768  CTCAACACAG CTGCTCAGGA CAAGCTCAGC
HWI-M01998:26:000000000-D2MKR:1:1102:22042:10802  CTCAACACAG CTGCTCAGGA CAAGCTCAGC
HWI-M01998:26:000000000-D2MKR:1:1102:13857:19231  CTCAACACAG CTGCTCAGGA CAAGCTCAGC
HWI-M01998:26:000000000-D2MKR:1:1101:20688:15446  CTCAACACAG CTGCTCAGGA CAAGCTCAGC

                                                 91
HWI-M01998:26:000000000-D2MKR:1:1102:21849:24863  TGTGTGTGTG TGTGTGT--- ----------
HWI-M01998:26:000000000-D2MKR:1:1101:18044:27768  TGTGTGTGTG TGTGTGTGTG TG--------
HWI-M01998:26:000000000-D2MKR:1:1102:22042:10802  TGTGTGTGTG TGTGTGTGTG TGTGTG----
HWI-M01998:26:000000000-D2MKR:1:1102:13857:19231  TGTGTGTGTG TGTGTGTGTG TGTGTGTGTG
HWI-M01998:26:000000000-D2MKR:1:1101:20688:15446  TGTGTGTGTG TGTGTGTGTG TGTGTGTGTG

                                                121
HWI-M01998:26:000000000-D2MKR:1:1102:21849:24863  ---------- ---------- -CTATGCGTG
HWI-M01998:26:000000000-D2MKR:1:1101:18044:27768  --------CG TGTGCGTGTG TCTATGCGTG
HWI-M01998:26:000000000-D2MKR:1:1102:22042:10802  --------CG TGTGCGTGTG TCTATGCGTG
HWI-M01998:26:000000000-D2MKR:1:1102:13857:19231  TGTGTG---- ----CGTGTG TCTATGCGTG
HWI-M01998:26:000000000-D2MKR:1:1101:20688:15446  TGTGTGTG-- ----CGTGTG TCTATGCGTG

                                                151
HWI-M01998:26:000000000-D2MKR:1:1102:21849:24863  TGTGTACGTG CAAGCAGGCT GGAGGAAGAG
HWI-M01998:26:000000000-D2MKR:1:1101:18044:27768  TGTGTACGTG AAAGCAGGCT GGAGGAAGAG
HWI-M01998:26:000000000-D2MKR:1:1102:22042:10802  TGTGTACGTG AAAGCAGGCT GGAGGAAGAG
HWI-M01998:26:000000000-D2MKR:1:1102:13857:19231  TGTGTACGTG CAAGCAGGCT GGAGGAAGAG
HWI-M01998:26:000000000-D2MKR:1:1101:20688:15446  TGTGTACGTG CAAGCAGGCT GGAGGAAGAG

                                                181
HWI-M01998:26:000000000-D2MKR:1:1102:21849:24863  GCCCTAAGGT AGAACAGAAC AGAGAACACA
HWI-M01998:26:000000000-D2MKR:1:1101:18044:27768  GCCCGAAGGT AGAACAGAAC AGAGAACACA
HWI-M01998:26:000000000-D2MKR:1:1102:22042:10802  GCCCTAAGGT AGAACAGAAC AGAGAACACA
HWI-M01998:26:000000000-D2MKR:1:1102:13857:19231  GCCCTAAGGT AGAACAGAAC AGAGAACACA
HWI-M01998:26:000000000-D2MKR:1:1101:20688:15446  GCCCTAAGGT AGAACAGAAC AGAGAACACA

                                                211
HWI-M01998:26:000000000-D2MKR:1:1102:21849:24863  AGAAAGGCAC GGCACAAGCT GTGGCCCGTC
HWI-M01998:26:000000000-D2MKR:1:1101:18044:27768  AGAAAGGCAC GGCGCAAGCT GTGGCCCGTC
HWI-M01998:26:000000000-D2MKR:1:1102:22042:10802  AGAAAGGCAC GGCGCAAGCT GTGGCCCGTC
HWI-M01998:26:000000000-D2MKR:1:1102:13857:19231  AGAAAGGCAC GGCACAAGCT GTGGCCCGTC
HWI-M01998:26:000000000-D2MKR:1:1101:20688:15446  AGAAAGGCAC GGCACAAGCT GTGGCCCGTC

                                                241
HWI-M01998:26:000000000-D2MKR:1:1102:21849:24863  AAGGGGCCCA TGGGAGACTC TCATGACGTG
HWI-M01998:26:000000000-D2MKR:1:1101:18044:27768  AAGGGGCCCA TGGGAGACTC TCATGACGTG
HWI-M01998:26:000000000-D2MKR:1:1102:22042:10802  AAGGGGCCCA TGGGAGACTC TCATGACGTG
HWI-M01998:26:000000000-D2MKR:1:1102:13857:19231  AAGGGGCCCA TGGGAGACTC TCATGACGTG
HWI-M01998:26:000000000-D2MKR:1:1101:20688:15446  AAGGGGCCCA TGGGAGACTC TCATGACGTG

                                                271
HWI-M01998:26:000000000-D2MKR:1:1102:21849:24863  GAACTATATT AATGCCAGTG CTGGACAGAA
HWI-M01998:26:000000000-D2MKR:1:1101:18044:27768  GAACTACATT AATGCCAGCG CTGGACAGAA
HWI-M01998:26:000000000-D2MKR:1:1102:22042:10802  GAACTACATT AATGCCAGCG CTGGACAGAA
HWI-M01998:26:000000000-D2MKR:1:1102:13857:19231  GAACTATATT AATGCCAGTG CTGGACAGAA
HWI-M01998:26:000000000-D2MKR:1:1101:20688:15446  GAACTATATT AATGCCAGTG CTGGACAGAA

                                                301
HWI-M01998:26:000000000-D2MKR:1:1102:21849:24863  CAAGGGGCAC CCCACACAGT ACTGGACTCT
HWI-M01998:26:000000000-D2MKR:1:1101:18044:27768  CAAGGGGCAC CCCACACAGT ACTGGACTCT
HWI-M01998:26:000000000-D2MKR:1:1102:22042:10802  CAAGGGGCAC CCCACACAGT ACTGGACTCT
HWI-M01998:26:000000000-D2MKR:1:1102:13857:19231  CAAGGGGCAC CCCACACAGT ACTGGACTCT
HWI-M01998:26:000000000-D2MKR:1:1101:20688:15446  CAAGGGGCAC CCCACACAGT ACTGGACTCT
```

331			
HWI-M01998:26:000000000-D2MKR:1:1102:21849:24863	AGAGCATAGG	GGGAAGTATA	ACATGTGATC
HWI-M01998:26:000000000-D2MKR:1:1101:18044:27768	AGAGTATAGG	GGGAAGTATA	ACATGTGATC
HWI-M01998:26:000000000-D2MKR:1:1102:22042:10802	AGAGTATAGG	GGGAAGTATA	ACATGTGATC
HWI-M01998:26:000000000-D2MKR:1:1102:13857:19231	AGAGCATAGG	GGGAAGTATA	ACATGTGATC
HWI-M01998:26:000000000-D2MKR:1:1101:20688:15446	AGAGCATAGG	GGGAAGTATA	ACATGTGATC
