# Supplementary material for: Automating microsatellite screening and primer design from multi-individual libraries using Micro-Primers
Source: Sci Rep. 2022 Jan 7;12:295. doi: 10.1038/s41598-021-04275-8 (PMC8741888; doi:10.1038/s41598-021-04275-8)
Supplement: Supplementary file 4 — Supplementary Information 4. [file 41598_2021_4275_MOESM4_ESM.pdf]

```
Alignment: /Mio/Papers/micro-primers/submission/verification/bats8_allele.phy
Seaview [blocks=10 fontsize=10 A4] on Mon Aug 30 16:50:49 2021

                                                  1
HWI-M01998:26:000000000-D2MKR:1:1102:17333:6605   GATCATAGGG TATCTGTCTT TCTCTGTCTT
HWI-M01998:26:000000000-D2MKR:1:1101:5833:22566   GATCATAGGG TATCTGTCTT TCTCTGTCTT
HWI-M01998:26:000000000-D2MKR:1:1102:22333:14528  GATCATAGGG TGTCTGTCTT CCTCTGTCTT
HWI-M01998:26:000000000-D2MKR:1:1102:12857:3802   GATCATAGGG TATCTGTCTT TCTCTGTCTT
HWI-M01998:26:000000000-D2MKR:1:1101:26400:22783  GATCATAGGG TGTCTGTCTT TCTCTGTCTT
HWI-M01998:26:000000000-D2MKR:1:1101:5279:15504   ----GATCAT AGGGTGTCTT TCTCTGTCTT
HWI-M01998:26:000000000-D2MKR:1:1101:18261:25389  GATCATAGGG TATCTGTCTT TCTCTGTCTT
HWI-M01998:26:000000000-D2MKR:1:1102:22508:18479  GATCATAGGG TATCTGTCTT TCTCTGTCTT
HWI-M01998:26:000000000-D2MKR:1:1101:11652:23167  GATCATAGGG TGTCTGTCTT TCTCTGTCTT
HWI-M01998:26:000000000-D2MKR:1:1101:19431:28091  GATCATAGGG TGTCTGTCTT TCTCTGTCTC

                                                 31
HWI-M01998:26:000000000-D2MKR:1:1102:17333:6605   GTTTCACTTC ACATTATGCC TCTAGGAACA
HWI-M01998:26:000000000-D2MKR:1:1101:5833:22566   GTTTCACTTC ACATTATGCC TCTAGGAACA
HWI-M01998:26:000000000-D2MKR:1:1102:22333:14528  GTTTCACTTC ACATTATGCC TCTAGGAACA
HWI-M01998:26:000000000-D2MKR:1:1102:12857:3802   GTTTCACTTC ACATTATGCC TCTAGGAACA
HWI-M01998:26:000000000-D2MKR:1:1101:26400:22783  GTTTCACTTC ACATTATGCC TCTAGGAACA
HWI-M01998:26:000000000-D2MKR:1:1101:5279:15504   GTTTCACTTC ACATTATGCC TCTAGGAACA
HWI-M01998:26:000000000-D2MKR:1:1101:18261:25389  GTTTCACTTC ACATTATGCC TCTAGGAACA
HWI-M01998:26:000000000-D2MKR:1:1102:22508:18479  GTTTCACTTC ACATTATGCC TCTAGGAACA
HWI-M01998:26:000000000-D2MKR:1:1101:11652:23167  GTTTCACTTC ACATTATGCC TCTAGGAACA
HWI-M01998:26:000000000-D2MKR:1:1101:19431:28091  ATTTCACTTC ACATTATGCC TCTAGGAACA

                                                 61
HWI-M01998:26:000000000-D2MKR:1:1102:17333:6605   TCAACGTTAT TGCAAATGGC AAGATTTCAA
HWI-M01998:26:000000000-D2MKR:1:1101:5833:22566   TCAACGTTAT TGCAAAATGC AAGATTTCAA
HWI-M01998:26:000000000-D2MKR:1:1102:22333:14528  TCCACGTTAT TGCAAATGGC AAGATTTCAA
HWI-M01998:26:000000000-D2MKR:1:1102:12857:3802   TCAACGTTAT TGCAAATGGC AAGATTTCAA
HWI-M01998:26:000000000-D2MKR:1:1101:26400:22783  TCAACGTTAT TGCAAATGGC AAGATTTCAA
HWI-M01998:26:000000000-D2MKR:1:1101:5279:15504   TCAACGTTAT TGTAAATGGC AAGATTTCAA
HWI-M01998:26:000000000-D2MKR:1:1101:18261:25389  TCAACGTTAT TGCAAATGGC AAGATTTCAA
HWI-M01998:26:000000000-D2MKR:1:1102:22508:18479  TCAACGTTAT TGCAAATGGC AAGATTTTAA
HWI-M01998:26:000000000-D2MKR:1:1101:11652:23167  TCCACGTTAT TGCAAATGGC AAGATTTCAA
HWI-M01998:26:000000000-D2MKR:1:1101:19431:28091  TCCACGTTAT AGCAAATGGC AAGATTTCAA

                                                 91
HWI-M01998:26:000000000-D2MKR:1:1102:17333:6605   TCATTTTCAT GGATGAGTTA TATCATACTG
HWI-M01998:26:000000000-D2MKR:1:1101:5833:22566   TCATTTTCAT GGATGAGATA TATCATACTG
HWI-M01998:26:000000000-D2MKR:1:1102:22333:14528  TCATTTTCAT GGATGAGATA TATCATACTG
HWI-M01998:26:000000000-D2MKR:1:1102:12857:3802   TCATTTTCAT GGATGAGATA TACCATACTG
HWI-M01998:26:000000000-D2MKR:1:1101:26400:22783  TCATTTTCAT GGATGAGATA TATCATACTG
HWI-M01998:26:000000000-D2MKR:1:1101:5279:15504   TCATTTTCAT GGATGAGATA TATCATACTG
HWI-M01998:26:000000000-D2MKR:1:1101:18261:25389  TCATTTTCAT GGATGAGATA TATCATACTG
HWI-M01998:26:000000000-D2MKR:1:1102:22508:18479  TCATTTTCAT GGATGAGATA TATCATACTG
HWI-M01998:26:000000000-D2MKR:1:1101:11652:23167  TCATTTTCAT GGAGGAGATA TATCATACTG
HWI-M01998:26:000000000-D2MKR:1:1101:19431:28091  TCATTTTCAT GGAGGAGATA TATCATACTG

                                                121
HWI-M01998:26:000000000-D2MKR:1:1102:17333:6605   CACACACACA CACACACACA CACACACACA
HWI-M01998:26:000000000-D2MKR:1:1101:5833:22566   CACACACACA CACACACACA CACACACAC-
HWI-M01998:26:000000000-D2MKR:1:1102:22333:14528  CACACACACA CACACACACA CACACAC---
HWI-M01998:26:000000000-D2MKR:1:1102:12857:3802   CACACACACA CACACACACA CACAC-----
HWI-M01998:26:000000000-D2MKR:1:1101:26400:22783  CACACACACA CACACACACA CAC-------
HWI-M01998:26:000000000-D2MKR:1:1101:5279:15504   CACACACACA CACACACACA C---------
HWI-M01998:26:000000000-D2MKR:1:1101:18261:25389  CACACACACA CACACACAC- ----------
HWI-M01998:26:000000000-D2MKR:1:1102:22508:18479  CACACACACA CACACAC--- ----------
HWI-M01998:26:000000000-D2MKR:1:1101:11652:23167  CACACACACA CACAC----- ----------
HWI-M01998:26:000000000-D2MKR:1:1101:19431:28091  CACACACACA CAC------- ---------C

                                                151
HWI-M01998:26:000000000-D2MKR:1:1102:17333:6605   C--ATGCGCA CACACACGAG CGCTCGCATC
HWI-M01998:26:000000000-D2MKR:1:1101:5833:22566   ---ATGCGCA CACACAAGAG CGCTCGCGTC
HWI-M01998:26:000000000-D2MKR:1:1102:22333:14528  -GCATGCGCC CACACACGTG CGCTCGCATC
HWI-M01998:26:000000000-D2MKR:1:1102:12857:3802   -GCATGCGCA CACACACGAG CGCTCGCATC
HWI-M01998:26:000000000-D2MKR:1:1101:26400:22783  ---ATGCGCA CACACACGAG CACTCGCATC
HWI-M01998:26:000000000-D2MKR:1:1101:5279:15504   ---ATATGCA CACACACGAG CACTCGCATC
HWI-M01998:26:000000000-D2MKR:1:1101:18261:25389  -GCATGCGCA CACACACGAG CGCTTGCATC
HWI-M01998:26:000000000-D2MKR:1:1102:22508:18479  -GCATGCGCA CACACACGAG CGCTCGCATC
HWI-M01998:26:000000000-D2MKR:1:1101:11652:23167  -GCATGCGCA CACACACGTG CGCTCGCATC
HWI-M01998:26:000000000-D2MKR:1:1101:19431:28091  CGCATGCGTA CACACACGTG CGCGCGCATC
```

```
                                          181
HWI-M01998:26:000000000-D2MKR:1:1102:17333:6605   ACATCCTCTT TATCCATTCA TCTGTTGAAG
HWI-M01998:26:000000000-D2MKR:1:1101:5833:22566   ACATCCTCTT TATCCATTCA TCTGTTGAAG
HWI-M01998:26:000000000-D2MKR:1:1102:22333:14528  ACATCCTCTT TATCCATTCA TCTGTTGAAG
HWI-M01998:26:000000000-D2MKR:1:1102:12857:3802   ACATCCTCTT TATCCATTCA TCTGTTGAAG
HWI-M01998:26:000000000-D2MKR:1:1101:26400:22783  ACATCCTCTT TATCCATTCA TCTGTTGAAG
HWI-M01998:26:000000000-D2MKR:1:1101:5279:15504   ACATCCTCTT TATCCATTCA TCTGTTGAAG
HWI-M01998:26:000000000-D2MKR:1:1101:18261:25389  ACATCCTCTT TATCCATTCA TCTGTTGAAG
HWI-M01998:26:000000000-D2MKR:1:1102:22508:18479  ACATCCTCTT TATCCATTCA TCTGTTGAAG
HWI-M01998:26:000000000-D2MKR:1:1101:11652:23167  ACATCCTCTT TATCTATTCA TCTGTTGAAG
HWI-M01998:26:000000000-D2MKR:1:1101:19431:28091  ACATCCTCTT TATCCATTCA TCTGTTGAAG

                                          211
HWI-M01998:26:000000000-D2MKR:1:1102:17333:6605   GACACCAGTG TTGCTTTTCT ATCTTGGCTA
HWI-M01998:26:000000000-D2MKR:1:1101:5833:22566   GATACCAGTG TTGCTTTTCT ATCTTGGCTA
HWI-M01998:26:000000000-D2MKR:1:1102:22333:14528  CACACCAGTG TTGCTTTTCT ATCTTGGCTA
HWI-M01998:26:000000000-D2MKR:1:1102:12857:3802   GACACCAGTG TTGATTTTCT ATCTTGGCTA
HWI-M01998:26:000000000-D2MKR:1:1101:26400:22783  GACACCAGTG TTGCTTTTCT ATCTTGGCTA
HWI-M01998:26:000000000-D2MKR:1:1101:5279:15504   GACACCAGTG CTGCTTTTCT ATCTTGGCTA
HWI-M01998:26:000000000-D2MKR:1:1101:18261:25389  GACACCAGTG TTGCTTTTCT ATCTTGGCTA
HWI-M01998:26:000000000-D2MKR:1:1102:22508:18479  GACACCAGTG TTGCTTTTCT ATCTTGGCTA
HWI-M01998:26:000000000-D2MKR:1:1101:11652:23167  GACACCAGTG TTGCTTTTCT ATCTTGGCTA
HWI-M01998:26:000000000-D2MKR:1:1101:19431:28091  GACACCAGTG TTGCTTTTCC ATCTTGGCTA

                                          241
HWI-M01998:26:000000000-D2MKR:1:1102:17333:6605   GCATACATAA TGCTGCAGTG AACCTAGAGG
HWI-M01998:26:000000000-D2MKR:1:1101:5833:22566   GCATACATAA TGCTGCAATG AACCTAGAGG
HWI-M01998:26:000000000-D2MKR:1:1102:22333:14528  GCATACATAA TGGTGCAGTG AACCTAGAGG
HWI-M01998:26:000000000-D2MKR:1:1102:12857:3802   GCATACATAA TGCTGCAGTG AACCTAGAGG
HWI-M01998:26:000000000-D2MKR:1:1101:26400:22783  GCATACATAA TGCTGCAGTG AACCTAGAGG
HWI-M01998:26:000000000-D2MKR:1:1101:5279:15504   GCATACATAA TGCTGCAGTG AACCTAGAGG
HWI-M01998:26:000000000-D2MKR:1:1101:18261:25389  GCATGCATAA TGCTGCAGTG AACCTAGAGG
HWI-M01998:26:000000000-D2MKR:1:1102:22508:18479  GCATACATAA TGCTGCAGTG AACCTAGAGG
HWI-M01998:26:000000000-D2MKR:1:1101:11652:23167  GCATACATAA TGGTGCAGAG AACCTAGAGG
HWI-M01998:26:000000000-D2MKR:1:1101:19431:28091  GCATACATAA TGGTGCAGAG AACCTAGAGG

                                          271
HWI-M01998:26:000000000-D2MKR:1:1102:17333:6605   CGAACGTATC TTTTCTGATC
HWI-M01998:26:000000000-D2MKR:1:1101:5833:22566   CGAACGTATC TTTTCTGATC
HWI-M01998:26:000000000-D2MKR:1:1102:22333:14528  CACACATATC TTTTCAGATC
HWI-M01998:26:000000000-D2MKR:1:1102:12857:3802   GGAACGTATC TTTTCTGATC
HWI-M01998:26:000000000-D2MKR:1:1101:26400:22783  CGAACGTATC TTTTCTGATC
HWI-M01998:26:000000000-D2MKR:1:1101:5279:15504   CGAACGTATC TTTTTTGATC
HWI-M01998:26:000000000-D2MKR:1:1101:18261:25389  CGAACGTGTC TTTTCTGATC
HWI-M01998:26:000000000-D2MKR:1:1102:22508:18479  CGAACGGATC ----------
HWI-M01998:26:000000000-D2MKR:1:1101:11652:23167  CCACGTATC- TTTTCAGATC
HWI-M01998:26:000000000-D2MKR:1:1101:19431:28091  CGCACGTATC TTTTCAGATC
```
